# Carrier-Assisted Nanomaterials and Microbial Dynamics in Advanced Wastewater Treatment: A Review

**DOI:** 10.3390/molecules31172991

**Published:** 2026-08-26

**Authors:** Zhongchuang Liu, Siu Hua Chang, Gilles Mailhot, Mohsen Taghavijeloudar, Valentin Romanovski

**Affiliations:** 1 Department of Environmental Engineering Technology, College of Power Engineering, Chongqing Electric Power College, No. 9, Electric Power Fourth Village, Jiulongpo District, Chongqing 400053, China; 2 Waste Management and Resource Recovery (WeResCue) Group, Chemical Engineering Studies, College of Engineering, Universiti Teknologi MARA, Cawangan Pulau Pinang, Permatang Pauh 13500, Penang, Malaysia; shchang@uitm.edu.my; 3 Institut de Chimie de Clermont-Ferrand, Université Clermont Auvergne—CNRS, F-63000 Clermont-Ferrand, France; 4 Department of Civil Engineering, Mazandaran Institute of Technology, Babol 4748161136, Iran; taghavi@mit.ac.ir; 5 Department of Materials Science and Engineering, University of Virginia, Charlottesville, VA 22904, USA; rvd9ar@virginia.edu

**Keywords:** nanomaterials, advanced treatment of wastewater, adsorption catalysis, microbial interactions, economic analysis

## Abstract

Nanomaterials (NMs) have shown broad application potential in wastewater deep treatment, but the actual application is constrained by some issues such as nanoparticle (NP) aggregation and poor recyclability. Different from previous comprehensive reviews, this article systematically synthesizes data from over 100 peer-reviewed studies (2012 to 2026) to review the preparation methods, purification mechanisms, and removal efficiencies for various pollutants, and the technical and economic feasibility of NMs, with an emphasis on carrier-assisted immobilization and NM–microbial aggregate interactions. To start with, the methods of preparation were roughly distinguished into two categories which were “top-down” and “bottom-up” methods. The advantages, disadvantages, and utilities of the physical, chemical, and eco-friendly methods of biosynthesis were investigated while paying particular attention to the function of the loading technique in preventing NP aggregation and improving recyclability. By using the technique of loading in the carrier, the growth of NPs could be restricted up to 2–50 nm. Secondly, seven basic mechanisms that underlie the process of removing pollutants by using NPs were explained: adsorption, catalytic degradation, ion exchange, surface complexation, antibacterial action, redox transformation, and waste recycling. Particular focus was placed on understanding the interactions between NMs, microbial aggregates, and extracellular polymeric substances in wastewater treatment systems. Extracellular polymeric substances (EPS) could capture >90% NMs and mitigate their toxicity. Once again, the removal efficiency and main influencing factors associated with different types of NMs, for the treatment of heavy metals, dyes, antibiotics, and pathogenic microorganisms were summarized. Removal efficiencies of the pollutants ranged from 70% to over 99%, but these values were strongly influenced by pH and matrix and often decreased substantially in real wastewater. The existing literature was used to classify the experimental substrates (single-solute systems, multi-solute synthetic systems, municipal wastewater, industrial wastewater, secondary effluent). The performance of NMs in different categories was compared, revealing the huge performance gap between ideal laboratory conditions and practical applications. Lastly, the economic viability of the methods based on the use of NMs for purifying water was assessed taking into consideration various factors such as raw materials’ prices, energy costs of the process of making materials, recyclability of the materials, and the possibility of introducing the use of NMs on a larger scale. Unlike existing reviews, this article aims to provide a quantitative mechanistic framework bridging the rational design, safe application, and engineering promotion of NMs in deep wastewater treatment.

## 1. Introduction

The 21st century marks the shift in the focus of water pollution regulation from just preventing the decline of water quality to ensuring water safety and improving the water environment [1,2,3,4]. Concurrently, increasing global water scarcity and more stringent wastewater discharge regulations have driven the continuous advancement of wastewater treatment technologies.

Nanomaterials (NMs) refer to materials that have at least one dimension in nanometer range (1–100 nm) or consist of them as constituents in the three-dimensional space [5,6]. Their extremely small dimensions endow them with unique physical and chemical properties significantly different from those exhibited by conventional bulk materials. Materials classified as NMs include both inorganic nanoparticles (e.g., metal nanoparticles such as Ag, ZnO, and CeO_2_) and carbon-based NMs (e.g., graphene oxide and carbon nanotubes), and nanometal oxides (e.g., TiO_2_ and Fe_3_O_4_). They are one of the most promising functional materials for wastewater treatment since NMs possess unique adsorption properties, the ability to photocatalytically decompose organic pollutants, and antibacterial behavior [5,6]. However, there are also some problems in the practical application of NMs. For example, dispersed nanopowders are often difficult to retain within treatment systems, prone to agglomeration that reduces their active surface area and performance, and challenging to separate and recover after use, thereby increasing the risk of secondary environmental contamination unfavorable for human health [5,6,7,8]. Thus, achieving the optimal compromise between high activity and usability of NMs has become a key problem on the road to their implementation.

In recent years, considerable research efforts have been devoted to addressing the challenges associated with the application of NMs in advanced wastewater treatment. Some relevant studies are as follows: (1) loading NMs on millimeter-scale porous carriers (polymer resin, activated carbon, minerals, and ceramic membrane) to form “milli-nano structure” composite materials, stabilizing nanoparticles (NPs) through the confined space of the carrier and ensuring fluid compatibility in the reactor [9,10]; (2) exploring the interaction interface between NMs and microbial aggregates such as activated sludge and biofilm in the treatment system of sewage, and revealing the regulation mechanism of extracellular polymeric substances (EPS) as a “dynamic barrier” on the toxicity mitigation and occurrence form of NMs [11,12,13,14]; (3) modifying the physical and chemical properties (hydrophilicity, pore size, and porosity) of the support layer of polyamide film composite membrane (TFC) to control the interfacial polymerization process accurately [15]. Most of the existing reviews either concentrated on the synthesis and inherent reactivity of NMs or separately on the ecological toxicity of NMs without establishing a comprehensive framework that combines material science with biological system responses in wastewater management. This article intends to fill this void by providing comprehensive coverage about NMs in advanced wastewater treatment, especially about the relationship between the design of the material and its biological interfaces.

Drawing upon both landmark foundational research and recent breakthrough studies, this article provides comprehensive coverage of NM synthesis approaches, methods of removal of pollutants and efficiency of waste treatment with emphasis on the role of carrier-assisted immobilization, green biosynthesis, and efficiency of treatments in wastewater of real composition. The present state of affairs and the limitations of nanotechnology-based wastewater remediation techniques are reviewed to establish a solid scientific basis for the sustainable development and implementation of these solutions. The methodology of the reference search is explained from the point of view of its completeness in Section 2. Section 3 provides a detailed overview of NM synthesis methods with an emphasis on the carrier-induced and environmentally friendly methods. Section 4 elaborates on the basic concepts that determine the nature of the process of wastewater treatment via using NMs. This presentation concentrates upon the relation between NMs and microbial aggregates. The successes in terms of the removal of hazardous substances, colors, drugs, and pathogenic microorganisms are presented in Section 4. At the same time, factors influencing the efficiency of NMs are discussed. In Section 5, a wide-ranging economic analysis of NMs is provided. In Section 6, future challenges in the development of nanotechnology-based techniques are highlighted. Finally, Section 7 contains some concluding remarks regarding the current state of affairs.

## 2. Literature Selection Methodology

This article is a narrative review. Well-defined keywords and databases were used to ensure transparency and reproducibility. The search for the relevant literature included various scientific databases such as the Web of Science, Scopus, PubMed, and China National Knowledge Infrastructure (CNKI). It also covered the discovery of studies related to research on NMs in wastewater treatment on a local and international level. The search process took place between January 2006 and December 2025, resulting in complementary updates in March 2026. The search keywords consist of the following terms and words: “nanomaterials” OR “nanoparticles” OR “nanocomposites” AND “sewage treatment” OR “advancement of sewage treatment” OR “water purification” AND “adsorption” OR “catalysis” OR “photocatalysis” OR “antimicrobial” AND “removal mechanism” OR “pollutant removal”. The results achieved were derived in accordance with Boolean operators (AND, OR).

The inclusion principles consist of: (1) peer-reviewed original and review papers published in English or Chinese; (2) exploration of NMs in wastewater treatment including all aspects such as preparation mechanisms, principles of operation and treatment efficiency; and (3) articles containing experimental data on the efficiency of removing pollutants as well as analysis of the mechanisms or economic efficiency. The exclusion criteria are: (1) conference abstracts, editorials, and non-peer-reviewed publications; (2) research not directly related to wastewater treatment based on NMs; (3) articles in which the experimental conditions are not fully reported; (4) articles without basic statistical analysis; and (5) repeatedly published literature.

The titles, abstracts and keywords were first screened for Chinese-language articles retrieved from CNKI. Articles which met the initial relevance criteria were retrieved in full text. They were read and analyzed in their original language. Chinese-language articles were included in this review. Key data and findings were extracted and incorporated into the review.

**Figure 1:**
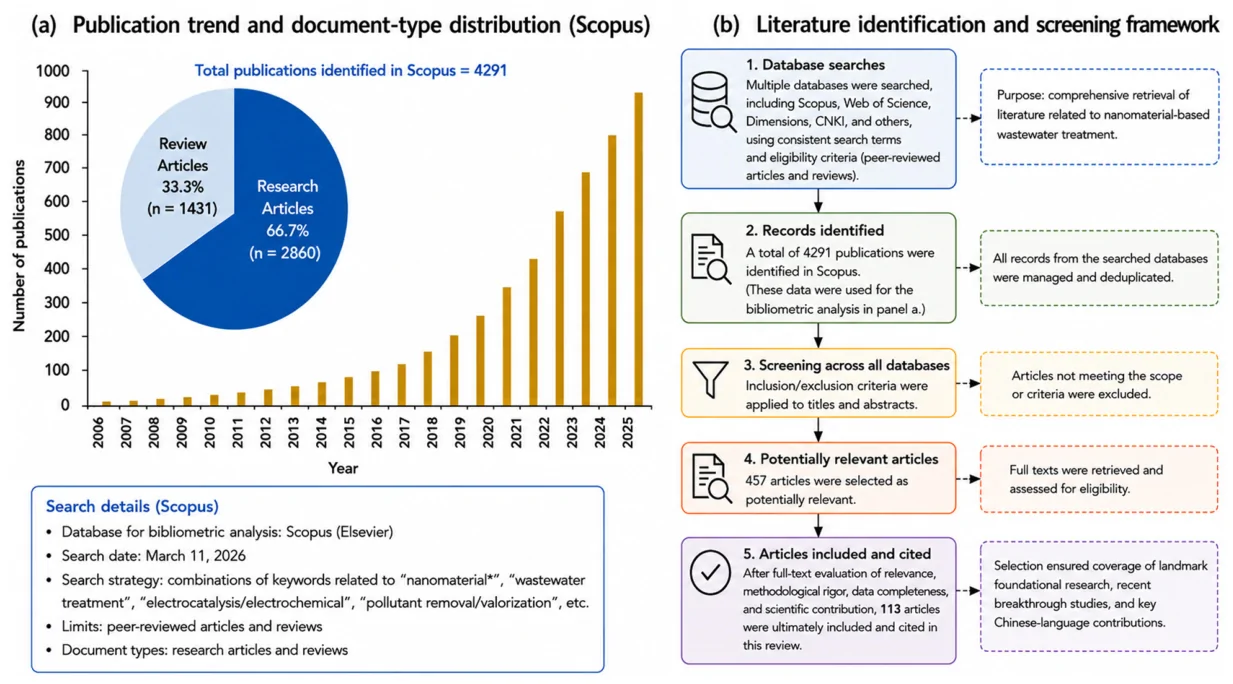
Bibliometric overview and literature-screening framework used in this review. (**a**) Annual Scopus publications from 2006 to 2025 and their distribution by document type. (**b**) Literature identification and screening across all databases, yielding 457 potentially relevant articles and 113 ultimately included and cited after full-text evaluation of relevance, methodological rigor, data completeness, and scientific contribution. Bibliometric data were retrieved from Scopus on 11 March 2026.

In the case of the Scopus database, the count of publications was 4291, which included 2860 research articles (66.7%) and 1431 review articles (33.3%) (Figure 1). The number of publications increased substantially from 9 in 2006 to 930 in 2025, indicating growing research interest in nanomaterial-based wastewater treatment technologies (Figure 1). The bibliometric data shown in Figure 1 were extracted from Scopus using the same keyword combinations as mentioned above, with the search restricted to peer-reviewed articles and reviews. The data were retrieved on 11 March 2026.

After applying the inclusion/exclusion criteria across all databases, 457 articles were chosen as potentially relevant references. After analyzing their relevance, methodological rigor, data completeness and scientific contribution, 113 articles were ultimately cited in this review. The bibliometric trend in Figure 1 is based on the retrieved data in the Scopus database; however, the references cited throughout this article are drawn from all databases searched and include landmark foundational research, recent breakthrough studies, and key Chinese-language contributions.

## 3. Preparation of NMs

The methods for preparing NMs mainly include laser ablation, vapor deposition, ball milling, plasma synthesis, hydrothermal/solvothermal synthesis, reverse micellar, co-precipitation, sputtering, and so forth [16,17,18,19,20]. More preparation methods of NMs are shown in Table 1. Preparation methods of NMs can be divided into two categories roughly. One is called the “top-down method”, and the other one is named the “bottom-up method” [16,17,18,19,20]. However, this dichotomy is overly simplistic and cannot fully encompass all methods. For instance, green synthesis and interfacial polymerization methods involve complex chemical and biological processes, combining elements of both or being self-contained systems. Therefore, a more effective approach is to conduct a multi-dimensional assessment based on their core driving forces and process characteristics. The top-down method breaks bulk materials to the nanometer scale by mechanical, thermal, or photolithographic techniques. Typical methods include ball milling, laser ablation, sputtering, arc discharge, and photolithography [16,17,18,19,20]. These methods are advantageous because of their simplicity, scalability, and limited solvent requirements [16,17]. However, they often exhibit poor control over particle size distribution and may generate structural defects or introduce impurities that adversely affect material performance [21,22,23]. For instance, the lattice distortion introduced by high-energy ball milling may serve as an active site or an electron-hole recombination center [21,22,23]. The ultimate effect highly depends on the material system and processing conditions, and this uncertainty poses challenges for its reliable application in the treatment of wastewater. The bottom-up approach generates NPs by assembling atomic or molecular precursors through chemical or physical pathways. The methods covered are numerous and diverse, including chemical vapor deposition, the sol–gel method, co-precipitation method, hydrothermal/solvothermal method, microemulsion method, template-assisted synthesis, spray pyrolysis, electrochemical synthesis, sonochemical synthesis, photochemical synthesis, the supercritical fluid method, molecular self-assembly, and plasma synthesis [24,25,26,27,28,29,30]. The abovementioned methods allow for effective control over NP size, shape and surface properties. Nevertheless, implementation of those methods may encounter certain challenges such as high cost of equipment, low production yields and complexity of removing surfactants after synthesis. More importantly, certain methods, such as the hydrothermal/solvothermal method, can potentially synthesize materials with high crystallinity, demonstrating superior stability, while for others, like chemical reduction, the residual stabilizers/terminal agents on the surface of the products may clog the active sites or fall off during use, becoming new pollutants. Therefore, merely focusing on precise control while neglecting the final application scenario of the materials may lead to suboptimal performance at high costs.

From an application perspective, different methods have their own advantages and disadvantages. Although ball milling and co-precipitation may appeal for large-scale production of raw materials due to their ease of implementation and low cost, the homogeneity of the resulting products is not adequate (Table 1). Whereas vapor deposition and hydrothermal synthesis can provide NMs with improved crystallinity and adaptable morphology, thus conforming to high-performance device criteria, the relatively high price of equipment and the need for strict regulation of the parameters of the process are key drawbacks (Table 1). Green synthesis has the advantages of eco-friendliness and excellent biocompatibility (Table 1), and green-synthesized NMs can be used in biomedical applications [25]. However, there are still problems in controlling the shape and size of the particles. Template-based synthesis and the reverse micelle technique allow achieving high accuracy in controlling size but face the issue of template removal and scaling of production (Table 1). Flame spray pyrolysis and spray pyrolysis are very suitable for industrial production owing to their high productivity (Table 1). Nevertheless, problems with agglomeration of particles and high temperature requirements still exist. In contrast, sonochemistry and photochemistry methods allow for fast NP formation under gentle working conditions, but do not provide much control over the particle properties. Therefore, hybrid synthesis approaches that incorporate different approaches have been developed to achieve the complementary advantages of different methods and counteract the limitations of every single method.

From the perspective of treating wastewater, the preparation methods of NMs must be matched with the application requirements. For example, for adsorption processes that require high dispersibility and long-term stability, materials synthesized by liquid-phase reduction or microemulsion methods may perform well, but their economic efficiency and scalability are bottlenecks. For photocatalytic degradation, the crystal phase, crystallinity and surface defects of the materials are the key factors determining their activity, which makes high-temperature treatment by calcination or hydrothermal methods more attractive. Therefore, the selection of preparation methods should not be isolated but must be comprehensively weighed against the properties of the target pollutants, the treatment process (adsorption/catalysis/antibacterial), and economic costs.

**Figure 2:**
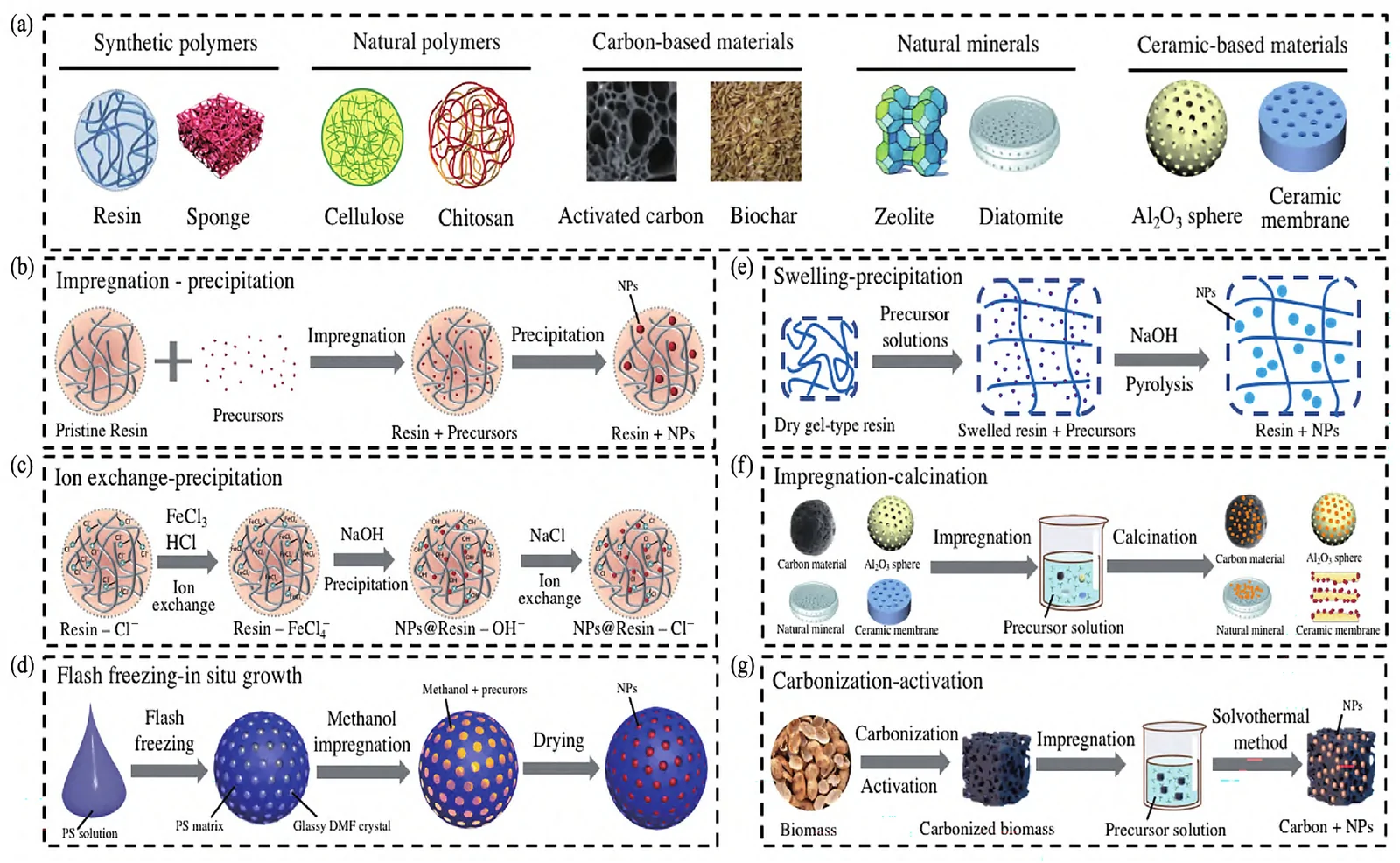
Types of carrier materials (**a**) and various physicochemical preparation methods for preparing nano-structured composites (**b**–**g**) ((**b**): impregnation–precipitation; (**c**): ion exchange–precipitation; (**d**): flash freezing in situ growth; (**e**): swelling–precipitation; (**f**): impregnation–calcination; (**g**): carbonization activation) [35].

To cope with the difficulties associated with low stability of the dispersed nanopowders that are used in reactors, many studies have integrated NMs with supports larger than millimeters in order to produce nanocomposites [35]. At present, these support materials can be broadly divided into four categories: polymer-based support, carbon-based support, natural mineral support, and ceramic-based support (Figure 2). Loading amounts of NPs on the four categories are 50~200 mg/g, 100~300 mg/g, 30~120 mg/g, and 80~250 mg/g, respectively [35]. In addition, NP sizes after loading of these categories are 5~30 nm, 10~50 nm, 20~50, and 5~10 nm, respectively [35]. Among them, polymer-based supports primarily comprise ion exchange resins and synthetic polymers, which are widely employed owing to their tunable surface properties and high loading capacities. The preparation methods are predominantly based on impregnation–precipitation and ion-exchange precipitation techniques, which promote uniform NM distribution and strong carrier–nanomaterial interactions (Figure 2). Such methods can achieve uniform dispersion of NPs in millimeter-scale supports and enhance the selective adsorption of target pollutants through the Donnan membrane effect. Carbon-based support materials include granular-activated carbon and biochar (Figure 2). Their preparation mainly adopts impregnation, calcination, or carbonization activation (Figure 2). After mixing biomass with metal salt precursors, the carbon matrix is formed by high-temperature pyrolysis and the NPs are confined in the carbon skeleton. This strategy can effectively inhibit the agglomeration of easily oxidized NPs, such as nano-zero-valent iron, and use the conductivity of carbon materials to enhance the electron transfer efficiency in the catalytic reaction. Natural mineral carriers include zeolite, diatomite and bentonite (Figure 2). Due to the stable physical pore structure and abundant surface active sites of natural minerals, impregnation–calcination is the core method to transform them into functional composites. This method has the advantages of low cost and abundant raw materials, but there is still room to improve the load uniformity and size control of NPs. Ceramic-based carriers are represented by alumina spheres and ceramic membranes (Figure 2). The sol impregnation–calcination method is the mainstream technical scheme. By immersing the carrier in the sol system containing titanium, cerium, manganese and other metals, and then calcining at high temperature, the nano oxide is firmly anchored to the surface and internal channels of the carrier. The high-gravity-assisted impregnation technology (HGI) developed in recent years has significantly improved the dispersion uniformity of NPs in the pores of ceramic carriers, so that the size of loaded particles can be controlled in the range of 5~10 nm [36,37,38,39,40]. HGI is a method that utilizes a rotating packed bed or rotating disk reactor to generate a supergravity field (centrifugal acceleration can reach hundreds to thousands of times that of earth’s gravity) to enhance liquid–solid mass transfer processes [36,37,38,39,40].

The preparation process of most carriers follows the three-stage path of “carrier pretreatment—nano precursor introduction—in situ conversion and fixation” (Figure 2). Precursor introduction mainly depends on impregnation, ion exchange, or physical mixing, while in situ conversion is achieved by chemical processes such as precipitation, pyrolysis, calcination, or reduction. Secondly, the confinement space is the core mechanism used to control the size and crystal form of NPs. The physical constraint of the carrier pore can effectively inhibit the excessive growth and agglomeration of NPs, so that the particle size is generally controlled in the range of 2~50 nm [37]. Especially when the pore size matches the size of NPs, the metastable crystalline form can be formed preferentially and exist stably [37]. In addition, the interaction between the carrier and NPs (such as electrostatic attraction and coordination) has a decisive effect on the firmness of the load [38,39]. The advantages of polymer matrix composites lie in their strong functional diversity and high selectivity. There have been many successful pilot and engineering application cases, but their structural stability and microplastic release risk in long-term operation need to receive continuous attention [40]. Carbon-based materials have a wide range of raw materials, low cost, and can achieve adsorption and catalytic functions in coordination [40]. However, biochar lacks a unified preparation standard and large-scale production process [40,41,42], which restricts its engineering promotion. Natural mineral reserves are rich and environment friendly, but the pore size distribution is uneven, and the loading of NPs is low [41,42]. A ceramic-based carrier has high mechanical strength and strong anti-fouling ability, which is suitable for integration in membrane separation and advanced oxidation coupling processes [41,42]. However, high-temperature calcination consumes more energy, and the equipment investment cost is high [41,42].

**Figure 3:**
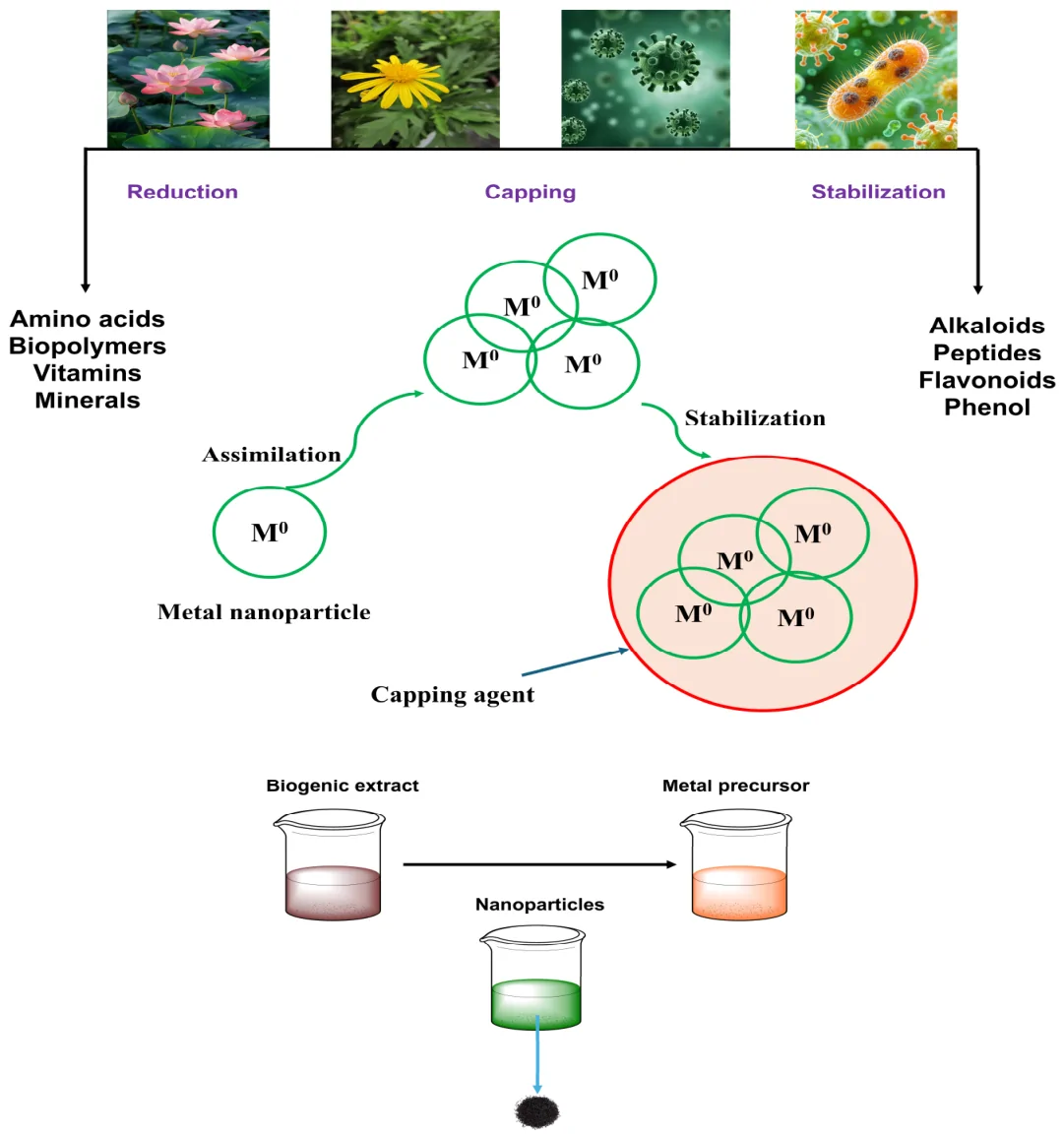
Biological synthesis of NMs.

In addition to physicochemical synthesis of NMs, biosynthesis is a common method. Biosynthesis is a method used to prepare metal or metal oxide NPs under mild conditions by using plant extracts, microorganisms (bacteria, fungi, yeast, algae, actinomycetes), and natural polysaccharides as reductants, stabilizers, and capping agents [43,44,45]. Its core is to avoid the use of toxic chemical reagents and high-energy consumption processes, and to achieve environmentally friendly production. First, the appropriate biological raw materials need to be selected. They include leaves, peels, seeds, flowers, and bark of plants. Microorganisms need to be amplified and cultured in the appropriate medium and collected extracellular secretions or cell lysates (Figure 3). Secondly, the biological extract is prepared. Then, a certain concentration of metal precursor salt solution (e.g., AgNO_3_, HAuCl_4_, FeCl_3_, ZnSO_4_, CuSO_4_, etc.) is mixed with the biological extract in proportion. Under the set pH (usually 4–9), temperature (room temperature to 80–100 °C), reaction time (minutes to hours), and stirring conditions, the biological molecules reduce metal ions to zero-valent atoms through electron transfer, and then nucleate, grow, and stabilize into NPs [43,44,45]. The color change of the solution during the reaction (such as the change of silver NPs from light yellow to brown) can be used as a preliminary indicator, and then pure NMs can be obtained by centrifugation, washing and drying (such as freeze drying or oven drying at 60–80 °C) [43,44,45]. Different biological sources have different emphases on methods. In addition, polysaccharide biomacromolecules represented by chitosan are usually dissolved in dilute acid solution, and then mixed with metal precursors to achieve complexation, reduction and spatial stability with the help of its rich hydroxyl and amino groups (Figure 3). pH value affects the ionization state and reduction ability of biomolecules, and alkaline conditions (pH 8–9) usually promote the deprotonation of phenolic hydroxyl groups to accelerate nucleation and obtain smaller particle sizes (Figure 3). In general, elevated temperature increases the rate of chemical reactions and promotes the formation of finer product particles. However, extreme temperatures can lead to considerable agglomeration and lower the uniformity of the formed products. The reaction time also needs to be carefully controlled, because too little time will result in incomplete reduction of the precursors and too much time may result in Ostwald ripening leading to the formation of large-sized particles. Moreover, the amount of the obtained products and the size distribution of the product particles depend on the concentration of metal salts used for the reaction as well as the proportion of the biological extracts used. Other parameters such as stirring speed, lighting conditions, and storage conditions have to be taken into account as well.

However, there are bottlenecks in practical engineering applications for biosynthetic methods Firstly, scaling up faces fundamental obstacles. The concentration of active ingredients in biological extracts fluctuates dramatically with plant species, growing seasons, soil conditions, and extraction processes, resulting in poor reproducibility of product particle size, morphology, and crystal phase between batches, which is particularly prominent in large-scale production. Even if microbial fermentation is used, there are still risks of gene drift, metabolic pathway changes, and bacterial contamination during the amplification and cultivation process, making stable supply of standardized bioreactors a challenge. Secondly, the complexity and uncontrollability of the reaction system are much higher than those of chemical synthesis. Biological extracts contain hundreds of compounds (polyphenols, flavonoids, proteins, polysaccharides, etc.), which synergistically or antagonistically affect the reduction kinetics and end-capping effect, making it almost impossible to precisely control the particle size distribution (such as controlling it within 5–20 nm) through conventional process parameters (pH, temperature, time). Additionally, the yield is generally low. Most biosynthetic NMs have a yield of only grams per liter of reaction solution at the laboratory scale, and the subsequent purification (removal of residual biomolecules) steps are cumbersome, significantly increasing the production and time costs per unit mass of material. Lastly, there are long-term stability issues. The surface of biosynthetic NPs is coated with an organic layer, which endows them with good dispersibility. Nevertheless, this organic layer is prone to detachment or degradation under actual wastewater treatment conditions (high ionic strength, extreme pH, oxidation conditions), leading to particle aggregation and loss of activity. Chemical synthetic materials typically have more stable inorganic ligands or shell protection.

## 4. Purification of Various Types of Wastewater

### 4.1. Fundamental Mechanisms

**Figure 4:**
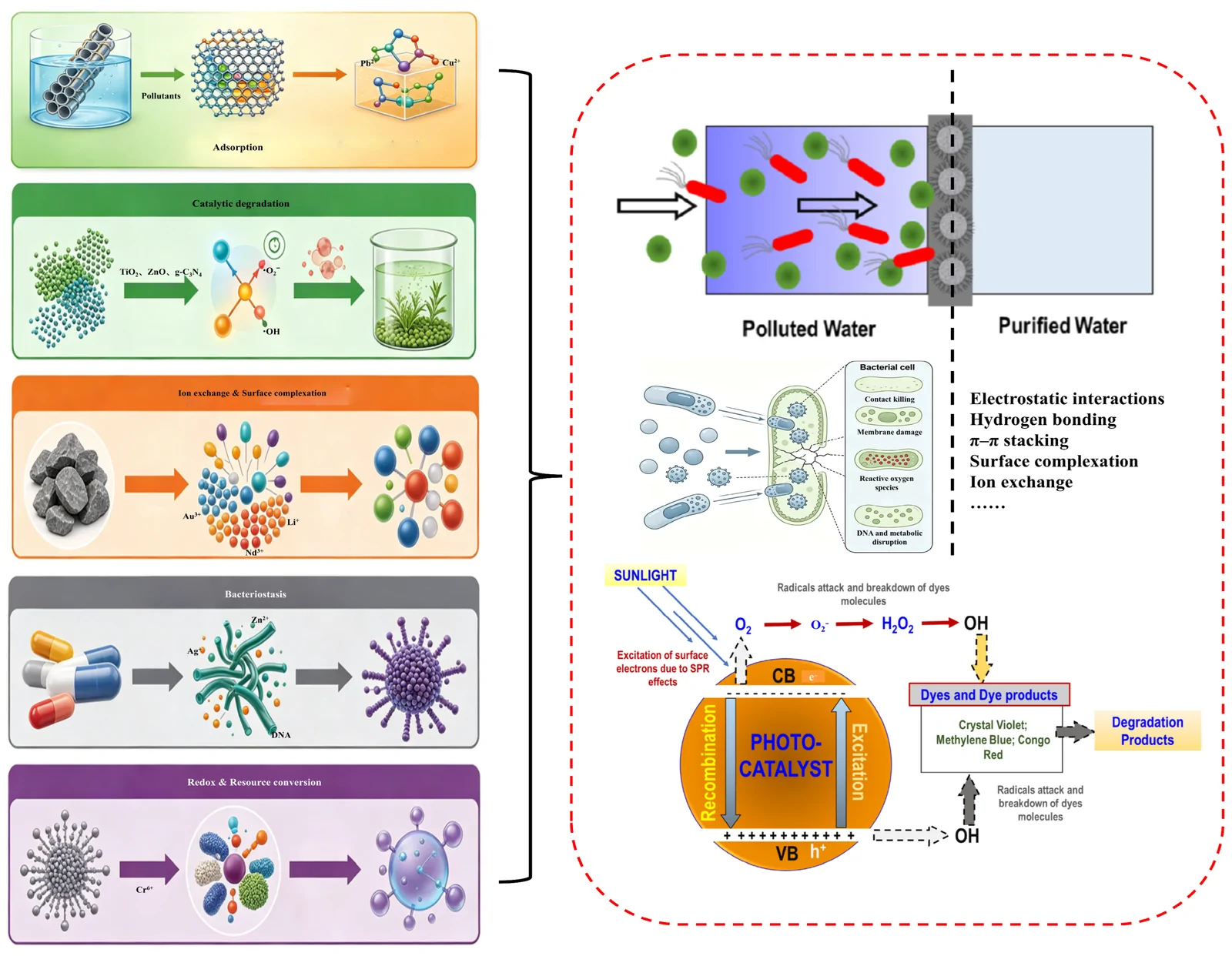
Mechanisms of NMs for removing pollutants from water.

The mechanisms of NMs for removing pollutants from water can be classified into the following categories: adsorption, catalytic degradation, ion exchange, surface complexation, bacteriostasis, redox, and resource conversion (Figure 4). Starting from the goals of environmental engineering, these mechanisms can be systematically classified into three categories based on their ultimate effects and outcomes: (1) physical separation (removal) from the aqueous phase; (2) chemical transformation (transformation/detoxification) of pollutants; and (3) complete destruction of organic pollutants (mineralization/permanent destruction).

Physical separation is the process by which pollutants are enriched from the aqueous phase onto the surface of NMs, without altering the chemical structure of the pollutants themselves. Its core mechanism is adsorption. Adsorption is one of the most widely used mechanisms of NMs [46,47]. It dominates as the concentration of pollutants is low (μg/L-mg/L level) and the water quality is relatively clean (low ion strength, low organic background). Carbon nanotubes (CNTs), graphene (GO) and reduced graphene oxide (rGO), metal organic frameworks (MOFs), mesoporous silica, and other materials have very high specific surface areas (up to 1000 m^2^/g) and rich active sites [48,49,50]. Pollutants in water can be adsorbed onto NMs through various mechanisms, including electrostatic interactions, hydrogen bonding, π-π stacking, surface complexation, and ion exchange [48,49,50]. In particular, charged surfaces of NMs contribute to adsorption of opposite-charged pollutants because of electrostatic attraction [48,49,50]. For instance, carboxyl and hydroxyl groups of the graphene oxide surface are protonated at a specific pH and, since they are negatively charged, can efficiently adsorb cations (e.g., Pb^2+^, Cu^2+^) (Figure 4). Aromatic rings present in organic pollutants (like dyes and antibiotics) are adsorbed on π-π electron clouds present on graphenes and CNTs, and the oxygen-containing functional groups form hydrogen bonds with pollutants. In addition, the hydroxyl groups on the surface of metal oxides (such as MnO_2_, TiO_2_, Fe_3_O_4_) form inner or outer sphere complexes with heavy metal ions [51,52,53,54,55]. For instance, As(III) can be specifically complexed by oxygen vacancies on the surface of CeO_2_ [56]. In zeolite, layered double metal hydroxides (LDHs), and other materials, exchangeable cations (such as Na^+^ and Ca^2+^) in the framework, exchange with heavy metal ions in the water to be removed [57,58]. Some studies have shown that surface functionalization with groups such as -NH_2_, -SH, and -COOH can significantly improve the adsorption selectivity and capacity [59,60,61,62]. For example, the adsorption capacity of mercaptan-modified MOF for Hg^2+^ was more than 1000 mg/g, and the adsorption capacity of Pb^2+^ on amino-functionalized mesoporous silica increased by more than three times [61,62]. There are some limitations for adsorption. Adsorption is only for the transfer of pollutants and does not achieve degradation. Subsequent desorption or immobilization treatment is required. In actual wastewater, coexisting ions (Ca^2+^, Mg^2+^, Cl^−^, SO_4_^2−^) and natural organic matter (NOM) compete for sites or block pores, resulting in a 30%-70% decrease in adsorption capacity. Under high-concentration pollutants, the adsorption sites rapidly saturate and the removal rate drops sharply. Furthermore, the regeneration process often requires strong acids/bases or organic solvents, which may damage the material structure and generate secondary waste liquid.

Permanent destruction is the preferred target for organic pollutant treatment, with the core being catalytic degradation, which thoroughly mineralizes organic pollutants (such as antibiotics, dyes, phenols) into CO_2_ and H_2_O through advanced oxidation processes. NMs can be utilized either directly as catalysts or as catalyst carriers to promote the degradation of organic pollutants through advanced oxidation processes, including photocatalysis, Fenton-like reactions, and persulfate activation [61,62]. Semiconductor NMs such as TiO_2_, ZnO and g-C_3_N_4_ generate electron hole pairs under light, and then generate reactive oxygen species (ROS) such as ·OH and ·O_2_^−^, which mineralize refractory organics such as antibiotics, dyes and phenols into CO_2_ and H_2_O (Figure 4). Previous research showed that the efficiency of tetracycline degradation catalyzed by defective TiO_2_ under visible light irradiation was more than 99% [63]. Nano-zero-valent iron (nZVI), Fe_3_O_4_, and CuO can catalyze the production of high concentrations of ·OH from H_2_O_2_ or persulfate. Studies have suggested that the Fe-N-C monatomic catalyst can initiate persulfate through a non-radical pathway, allowing for effective destruction of sulfonamide antibiotics in different pH conditions [64,65,66,67,68,69,70]. Moreover, nitrogen-doped carbon-supported monatomic Fe (Fe-SA/NC) has been proven to produce singlet oxygen (^1^O_2_) leading to an environmentally friendly and selective removal of organic pollutants. The advantage of catalytic degradation is that all pollutants are partially or completely mineralized [64,65,66,67,68,69,70]. Thus, it is possible to convert it into inorganic small molecules such as CO_2_ and H_2_O, rather than simply transferring pollutants from the aqueous phase to the solid phase. It must be mentioned that “complete mineralization” is not always possible, especially in the case of complex organic molecules that can form intermediate products, which might be more mobile or hazardous than the original pollutants during the process. Hence, while assessing the catalytic degradation efficiency, it is essential to consider not only the removal rate of the pollutants but also the generation of ROS, its quantum efficiency, photon efficiency, mineralization studies, the products of transformation, and catalyst stability. In addition, problems related to catalyst recovery, photocorrosion, and the toxicity of by-products need to be solved. The generation of toxic by-products during catalytic degradation is one of the key bottlenecks restricting the practical application of NMs. Research has shown that although advanced oxidation processes catalyzed by NMs can effectively degrade organic pollutants, pollutant molecules often do not completely mineralize into CO_2_ and H_2_O at once, but undergo a series of intermediate conversion steps [65,67,68,69,70]. During this process, intermediate products with higher toxicity than the parent compound may be generated, posing a risk of secondary pollution. For example, TiO_2_ photocatalytic breakdown of pentachlorophenol generates toxic by-products [67,68]. In addition, some carbon-based NMs undergo decomposition during advanced oxidation processes, and their dissolved components exhibit acute toxicity [69,70]. In response to the above issues, researchers have developed various strategies for assessing and regulating the toxicity of by-products in recent years [65,67,68,69,70]. At the level of analytical methods, technologies such as liquid chromatography–mass spectrometry (LC-MS) and gas chromatography–mass spectrometry (GC-MS) have been widely used for identifying degradation intermediates and deducing degradation pathways [65,66,67]. On this basis, quantitative structure activity relationship prediction software such as Ecological Structure Activity Relationships (ECOSAR) can systematically evaluate the aquatic ecological toxicity of intermediate products [68,69]. Meanwhile, biological testing methods such as *Daphnia magna*, zebrafish, and seed germination can be used to verify the comprehensive toxicity of the treated effluent [67,68,69,70].

Ion exchange mainly occurs in NMs with a layered or porous structure, such as zeolites, LDHs, and MOFs [71,72,73]. The mechanism of ion exchange is that the counterions in the material skeleton (e.g., Na^+^, K^+^, Mg^2+^) are replaced by heavy metal ions in the wastewater [71,72,73]. This process has high selectivity and is especially suitable for the recovery of low-concentration heavy metals and key metals (such as Li^+^, Nd^3+^, Au^3+^) (Figure 4). For example, the selective adsorption efficiency of the BioMOF@SWCNT composite material for Au^3+^ through sulfur-containing ligands was more than 98% [74]. Surface complexation occurs more widely in metal oxides and organic framework materials. Pollutant ions form covalent coordination bonds with surface hydroxyl or organic ligands, which belong to chemical adsorption [75,76,77,78]. This process is usually irreversible, which is suitable for the immobilization of highly toxic heavy metals. The ion exchange capacity is limited by the number of exchangeable sites within the skeleton, and the selectivity for non-target competing cations (Na^+^, Ca^2+^) is poor, requiring functional modification to enhance specificity.

For microbial pollution in water, NMs can achieve high-efficiency bacteriostasis through metal ion release, physical damage, photothermal/photodynamic synergy, and quorum-sensing inhibition [79,80,81,82]. This belongs to the “inactivation” of biological pollutants. NPs containing Ag, CuO, and ZnO can slowly release Ag^+^, Cu^2+^, and Zn^2+^, which can penetrate the cell membrane, and bind with sulfur-containing protein and DNA, blocking metabolism and replication (Figure 4). The killing rate of Ag-NPs to *Escherichia coli* and *Staphylococcus aureus* was close to 100% [80]. What is more, CNTs and graphene nanosheets have sharp edges, which can directly cut the bacterial cell wall and cause content leakage [76]. The physical antibacterial effect of GO and CNT membranes does not depend on ion release, so they are more resistant to drug resistance. According to previous research, under near-infrared light irradiation, TiO_2_/MoS_2_/polydopamine (PDA) composite nanorods produced local high temperature and ROS, which could kill more than 99% of bacteria and disintegrate biofilm in 10 min [83,84,85,86,87,88]. Additionally, CeO_2_ nanoenzymes can interfere with quorum-sensing signal molecules of bacteria and prevent biofilm formation. For example, CeO_2_-coated plastic containers reduced *Pseudomonas aeruginosa* biofilm by 100% [87,88]. However, uncontrollable ion release leads to limited antibacterial lifespan, and the leaching of metal ions may inhibit functional bacterial communities (such as nitrifying bacteria) within the system.

Chemical transformation aims to change the chemical valence state or existing form of pollutants in order to reduce their toxicity and mobility, or achieve resource recovery. This category mainly includes oxidation-reduction conversion and co-precipitation. The NMs assist the process of conversion of highly toxic and mobile metallic ions of high valence into lower valence or zero-valent metallic states, allowing the reduction of the risk of environmental pollution in the process of the recovery of useful metal resources. Some publications indicate a 99% reduction of Cr(VI) within a time of 30 min, which leads to the reduction of its toxicity considerably. In addition, it has been shown that N-doped Ti_3_C_2_T_x_ MXene (a two-dimensional transition metal carbide) can achieve 99.5% selective adsorption of Pb^2+^ at a low 1.5 V voltage with a stability of five cycles at the level of 97%. Another study showed that the Cd^2+^/Zn^2+^ adsorbed on mesoporous zeolite was transformed into a Cds-ZnS heterojunction photocatalyst by in situ vulcanization, which realized the dual functions of heavy metal recovery and hydrogen production [89]. Similarly, cellulose nanofibers adsorbed Cu^2+^ and restored it to Cu-NPs, which could be used for antibacterial methods or catalysis [90].

### 4.2. Interactions with Microbial Aggregates and EPS

**Figure 5:**
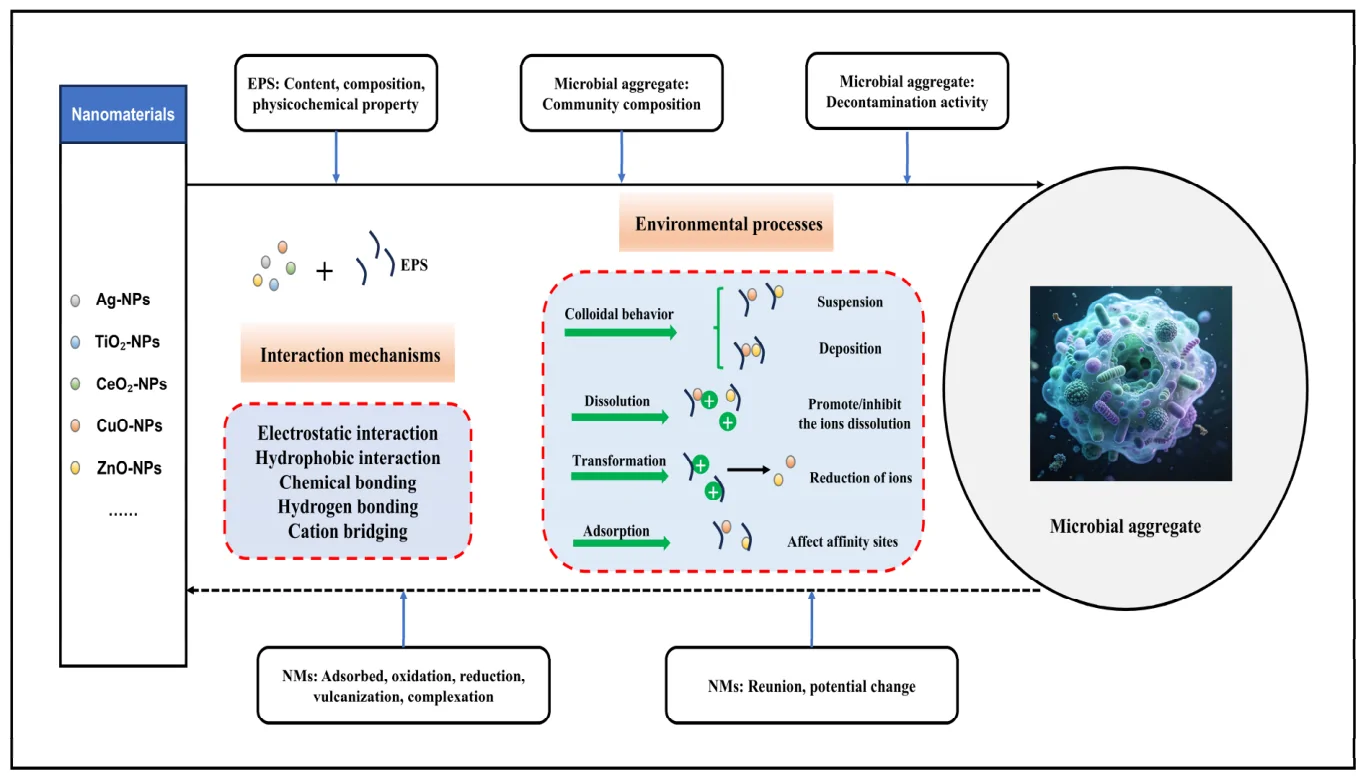
Interaction between NMs and microbial aggregates.

Microbial aggregates are three-dimensional structures made up of microbial cells that are held together with the help of extracellular polymeric substances (EPS), which allows them to adsorb certain substances effectively [88,89,90]. Some research showed that under certain conditions (for example, with the presence of carbon nanotubes or metal oxide NPs in neutral pH, low ionic strength synthetic wastewater), EPS could capture more than 90% of NMs as a result of the following processes: electrostatic interactions, coordination and hydrogen bonds [88,89,90] (Figure 5). In addition, this efficiency of adsorption was situation-specific, depending on the nature of NMs, size of particles, their surface charge, wastewater properties (pH, ionic strength, organic materials) as well as properties of microbial aggregates [88,89,90]. However, it should be noted that this adsorption efficiency (reported in the literature to exceed 90%) is not universally applicable, but highly dependent on the type of NMs (such as carbon based, metal oxide), particle size, surface charge, wastewater chemical properties (such as pH, ionic strength, organic matter content), as well as the morphology and surface properties of microbial aggregates. Some small-sized NMs (<50 nm) can enter the cell through membrane pores or endocytosis, and the ions dissolved from metal NMs can also enter the cell through specific channels [90,91]. However, NMs with a large particle size or high polymerization state are more easily captured by the EPS layer [90,91].

EPS, as an important component of microbial aggregates, serves as a “dynamic barrier” and critical reaction interface between NMs and microbial cells. The protein, polysaccharide, humus, and nucleic acid components in EPS contain abundant functional groups (such as carboxyl, hydroxyl, phosphate) [90,91], which can capture NMs through complexation, precipitation, or electrostatic attraction, thereby alleviating the direct toxicity of NMs to internal microbial cells to a certain extent (Figure 5). It has been shown in studies that protein and carboxylic acid functional groups of EPS are sites for the binding of NMs. For example, 98% of CeO_2_-NPs were trapped on the EPS or cell surface in the sequencing batch biofilm reactor (SBBR), and only a small amount entered the cell interior [84,88,89,90,91]. However, this capture may also alter the surface chemical state, aggregation behavior, and reactivity of NMs. At the same time, the presence of NMs can also stimulate microorganisms to secrete more EPS as a stress response. Although this may enhance the adsorption of pollutants, it may also increase mass transfer resistance and hinder the diffusion of substrates and oxygen to microbial cells.

NM exposure can change the content, composition and physicochemical properties of EPS (Figure 5). For example, Ag-NPs can bind to loose and tightly bound EPS, affecting their adsorption sites; TiO_2_-NPs can cause the red shift of protein fluorescence spectrum in EPS, promote the degradation of protein and polysaccharide, and even reduce its content at high concentration; the short-term exposure of CeO_2_-NPs will increase the content of polysaccharide and protein, while the long-term exposure will increase the content of polysaccharide and protein, and change the functional groups of EPS and flocculation performance. These changes further affect the surface charge, morphology, sedimentation, and rheology of microbial aggregates. For example, TiO_2_-NPs have been reported to increase sludge surface roughness and reduce flocculation efficiency, whereas CNTs can promote the accumulation of polysaccharides and proteins within the outer layer of granular sludge, thereby enhancing structural compactness and resistance to toxic stress. The viscosity of sludge decreased at low concentrations of CeO_2_-NPs and increased at high concentrations. Furthermore, NM exposure can also change the community composition of microbial aggregates (Figure 5). CuO-NPs increased the proportion of Proteus and acidogenic bacteria in biofilm, decreased Bacteroides, and increased the abundance of heavy metal-resistant bacteria (such as *Trichomonas* spp.) [85,86,87]. Under CeO_2_-NP exposure, ammonia oxidation-related bacteria (*Rhodofexa*, *Pseudoclavibacter*) decreased, some nitrifying bacteria (*Devosia*, *Salinibacterium*) increased, *Nitrospira* decreased, and denitrifying bacteria (*Citrobacter*, *Simplicispira*, etc.) increased [88,89,90]. CNTs can improve the abundance of methanogens and fermentation-related bacteria. Zero-valent iron NPs promoted the growth of hydrogenotrophic methanogens [90]. The attitude of microbe populations towards NMs is influenced by their location in the process system. Thus, a clear understanding has not yet been formed, which means that additional research under other microenvironment conditions is necessary. NMs can inhibit the removal efficiency of nitrogen and phosphorus by microbial aggregates (Figure 5). For instance, 50 mg/L ZnO-NPs reduced the total nitrogen removal rate from 81.5% to 70.8%, and the phosphorus concentration in the effluent increased significantly; CeO_2_-NPs (10 mg/L) inhibited ammonia oxidation and denitrification, and reduced the activities of key enzymes (such as nitrate reductase); CuO-NPs can improve the removal rate of total nitrogen, but reduce the phosphorus removal efficiency and increase the production of N_2_O. Ag-NPs inhibited nitrification activity and reduced the number of ammonia-oxidizing bacteria [90,91]. In addition, NMs can stimulate EPS production, which may enhance pollutant adsorption but also increase mass transfer resistance and hinder pollutant diffusion to microbial cells, thereby exerting a complex influence on overall treatment efficiency. The impact of NMs on microbial community structure is complex and selective, and its consequences cannot be simply classified as “beneficial” or “harmful”. It must be evaluated in conjunction with their specific effects on system treatment functions. The inhibitory or promoting effects of different types of NMs on different functional bacterial communities vary significantly.

The assessment of microbial toxicity for NPs must take into account not only the inhibition rate of one kind of bacteria but also the long-term effects of NPs on the functioning of the entire microbial ecosystem (including carbon, nitrogen, and phosphorus cycles), as well as possible risks for the quality of wastewater and sludge disposal. Transcriptomic studies showed the ability of ZnO NMs to regulate the expression of the genes involved in nitrogen metabolism, which is indicative of the ways of their action [83]. It should be noted that the reaction of microorganisms to NPs is dependent on their location, and there are no unified ideas regarding this matter at present. More research is needed under various microenvironment conditions.

### 4.3. Removal Efficiency by Pollutant Type

NMs are known to be highly effective and capable of removing various pollutants from wastewater. NMs employ their unique properties, such as high surface area and abundance of active sites, to destroy harmful substances, including heavy metals, organic dyes, drug residues, and pathogenic microbes, in a very cost-effective way. Various technical parameters associated with the removal of pollutants with NMs vary in terms of efficiency and diversity with regard to the specifics of the reduction process (Table S1). In general, NMs show great removal efficiency for various substances when operating under optimal conditions (Table S1). To estimate NMs’ capacity in removing different pollutants, this report presents the results obtained while conducting 73 experiments, described in Table S1. It has been determined from analysis that carbon-based NPs, specifically MWCNTs, GO, and their mixtures, can eliminate metals up to about 80–99% (Pb^2+^, Cd^2+^, Cu^2+^) at a pH value of 4 to 8 within 5–120 min of contact time (Table S1). Metal oxides work via a combination of adsorption and photodegradation processes with the efficiency of the removal of dyes and antibiotics anywhere from 70 to 96% (Table S1). However, such processes usually require much higher reaction times. NPs based on silver can eliminate >99% of pathogens, but the removal rates of metals fluctuate quite a lot (64–99.7%) (Table S1). It is worth noting that over 90% of the studies in Table S1 used single solute synthetic wastewater, with only a few experiments involving multi-solute coexistence systems, and studies using actual municipal or industrial wastewater are extremely rare.

The information provided in Table S1 indicates that NMs have great potential for the removal of many types of contaminants from water. These pollutants include not only heavy metals but also dyes, pharmaceutical compounds, pathogenic organisms, nutrients, and emerging pollutants. However, when it comes to comparing different NMs, there are great differences in removal mechanisms, conditions, retrieval efficiency, and degree of practical applicability. For example, carbon NMs, such as graphene oxide, reduced graphene oxide, CNTs, and their composites, show high adsorption capabilities when it comes to heavy metals and organic pollutants. This ability can be attributed to the exceptional characteristics of these NMs, which include the high specific surface area, presence of many oxygen-containing functional groups, and strong π-π interactions with aromatic substances. Most of the time, up to 90% of pollutant particles can be caught in a short amount of time. However, while removal is effective, it must be noted that the pollutants remain active. The substances are simply moved from the liquid to the solid phase.

Metal oxide NPs such as TiO_2_, ZnO, CuO, Fe_3_O_4_, and Fe_2_O_3_ have more versatility due to their combination of adsorption, photocatalytic degradation, oxidation, and reduction capacities. The TiO_2_-based systems are studied more than other materials due to their chemical stability and powerful oxidation actions. Their limitation of the use of ultraviolet radiation, however, makes their practical implementations impossible with sunlight. Even though the addition of dopants and forming composites have achieved visible-light activation, catalyst deactivation, electron-hole recombination, and recovery issues still remain in practice.

Out of all the mentioned materials, silver-based NMs have the greatest effect on antibacterial activity as they almost always achieve complete bacterial elimination. Their efficiency in killing microorganisms is based on many other processes such as the release of ions, cell membrane destruction, causing oxidative stress, or damaging DNA. However, the mass introduction of silver NPs into production is complicated by the problems with the release of silver ions, possible ecotoxicity, and high costs of this material.

NMs based on iron, including nano-zero-valent iron (nZVI), seem to be very promising candidates for practical applications in wastewater treatment. Apart from possessing high removal efficiency towards heavy metals, iron-based NMs have the ability to detoxify hazardous pollutants due to their capability to conduct reduction reactions, obtaining complete removal and detoxification of contaminants in the same process. Moreover, iron-based materials are much cheaper than noble metal-based NMs and are often easily separable by magnetic means. However, the major drawbacks of these materials are rapid oxidation, particle aggregation, and loss of activity during recycling.

Surprisingly, composite NMs seem to be more efficient than single-component NMs due to the synergy of their functional components. Materials like titanium dioxide–graphene oxide (TiO_2_–GO), multi-walled carbon nanotubes/polyaniline/magnetite (MWCNTs/PANI/Fe_3_O_4_), and chitosan–TiO_2_ demonstrate excellent properties of removal, wide operating ranges of pH and stability of usage. This indicated that further development of NMs should be based on the design of complex multifunctional systems instead of single-component systems.

As evidenced in Table S1, the high removal efficiencies highlighted the significant potential of the NMs explored in this paper, but it is important to be cautious when comparing efficiencies across different studies since most assessments have been conducted with the use of synthetic wastewater under controlled laboratory conditions. In most cases, the experimental systems do not reflect the true nature of real wastewater, where the presence of competing ions and organics, solid particles, and microorganisms disturbs pollutant removal processes and significantly reduces the efficiency of treatment. Therefore, there is a risk that the reported very high efficiencies obtained in laboratory conditions can overestimate the actual performance in the field.

Another important drawback of the literature is that there are no uniform testing methods. The differences in experimental conditions employed for pollutant concentration, reactor type, and metrics applied for performance measurements make comparisons difficult. It is vital that future work focuses on the adoption of standard evaluation procedures, assessing long-term performance, running experiments in the continuous-flow mode, and conducting pilot tests to enable comparison and aid commercial application of technologies.

In general, it appears from Table S1 that there is no NM that performs well in all forms of polluted wastewater. Therefore, the selection of the relevant material should be determined by the properties of the waste pollutant, treatment objectives, conditions of operation, and requirements for recovery, safety, and economy. It is expected that the future will see a trend towards the creation of multifunctional composite NMs capable of performing destruction, recovery, and adsorbing of pollutants all at once.

When evaluating the removal performance of NMs for pollutants, it is necessary to make stricter distinctions between their categories. There are significant differences in the structural characteristics, mechanism of action, adsorption kinetics, and thermodynamic behavior of NMs of different categories. According to existing research, NMs can be roughly divided into carbon-based loaded metal NPs, metal organic frameworks, MXenes, single atom catalysts, nanocomposite materials, and polymer loaded NMs [5,6,7,8]. The removal of heavy metals and organic pollutants by carbon-based loaded metal NPs usually follows a quasi-second-order kinetic model and Langmuir isotherm adsorption model, indicating that the adsorption process is dominated by chemical adsorption [88,89,90,91,92]. The adsorption characteristics of MXene materials are commonly emphasized by the Langmuir isotherm model and quasi-second-order kinetic model which supports the theory of predominance of chemical adsorption [88,89,90,91,92]. The behavior of the composites is often complex involving the interactions of many mechanisms at once [88,89,90,91,92]. It is worth noting that in recent years, polymer loaded materials based on natural polysaccharide chemical modification have shown excellent performance in heavy metal removal. For example, the sodium salt adsorbent (Na CL-SSH) prepared from the polysaccharide water gel extracted from the seeds of *Salvia spinosa*, which was cross-linked by citric acid, and then saponified by NaHCO_3_, had an adsorption capacity of 277.78 mg/g for Cd(II) (Langmuir model fitting value), and an experimental value of 235.71 mg/g [91]. An analogous technique was adopted in modifying mucilage in Artemisia vulgaris seeds [89]. The polysaccharide was modified by an esterification process through succinic anhydride and then converted to its sodium salt form (SSAVH) which exhibited adsorptivity of 263.15 mg/g for deionized water and 243.90 mg/g for Cd^2+^ ions [89]. In the same way, hydroxyethyl cellulose was modified by maleic anhydride and then converted into sodium salt form (HEC-MAL Na) which had an adsorptivity of 277.77 mg/g for Cd(II) [88].

According to the Langmuir model and the quasi-second-order kinetic models, the adsorption of various types of NMs indicates that chemical absorption and monolayer coverage are typical processes for most systems. Negative values of thermodynamic parameters (ΔH° < 0, ΔG° < 0) indicate that the process is exothermic and spontaneous. Nevertheless, the differences between different materials in terms of the adsorption performance, time required to reach equilibrium, selectivity, and regeneration define their different applicable scenarios.

### 4.4. Influencing Factors

Some important factors that influence the purification efficiency of NPs in wastewater are the pH, temperature, quantities of the adsorbent, initial concentration of pollutants, presence of other ions, contact time, and intensity of light as well as wavelength of irradiation [65,66,67,68,69,70]. The pH value is one of the most important parameters affecting the performance of NP adsorbents. The pH influences the surface charge of NPs and the form of pollutant. When the pH of the solution is lower than pH_pzc_, the surface of the NPs carries a positive charge, which is beneficial for the adsorption of anionic pollutants such as dyes and oxygen-containing anions [70]. Alternatively, the surface possesses a negative charge that facilitates the adsorption of cationic pollutants (like heavy metal ions) when the pH is above the pH_pzc_. In the case of carbon-based NMs, the effect of pH is observed when the surface functional groups (like the carboxylic and hydroxyl groups) undergo protonation/deprotonation. This, in turn, alters the driving forces for adsorption processes (electrostatic forces, hydrogen bonds, and π-π interactions). It is important to note that pH also influences the stability of dispersion of NPs [70]. When pH approaches its point of zero charge, absolute values of Zeta potential decrease, which causes weakening of electrostatic repulsion between particles and makes the particles prone to aggregation [70]. Furthermore, under extreme pH, metal ions can create hydroxide precipitates, thus giving the impression of a high removal rate, which is actually a precipitation mechanism, not an adsorption type and must be understood [70]. That is why the optimal pH range for different NPs depends on the material and must be calculated accordingly to the pH_pzc_ of the NPs and characteristics of the target pollutant. For heavy metal ions, the pH of the solution affects the degree of hydrolysis and the existing form [70]. Under acidic conditions, H^+^ competes with metal ions for adsorption sites. On the other hand, metal ions may form hydroxide precipitation under alkaline conditions. It was observed that the optimal pH needed for Pb(II) adsorption using GO-Gd_2_O_3_ was 4, whereas the maximum adsorption capacity using rGO was achieved for both Pb(II) and Cd(II) ions at the same pH level [71,84]. As for Zeolitic Imidazolate Framework-8-epigallocatechin gallate (ZIF-8-EGCG), the pH affects the protonation of the surface functional groups, and influences the selective adsorption of metals. The temperature acts as another factor that has a direct impact on both the dynamics and thermodynamics of the adsorption process [76,77]. In most cases, it has been shown that adsorption efficiency grows with growing temperature since this reaction appears to be endothermic. The optimum temperature for MWCNTs to adsorb As(V) was found to be 303 K (30 °C) at which the adsorption capability was 415.8 mg/g [76,83,84,85,86,87]. The increase in temperature enhances the speed of diffusion as well as the kinetic characteristics of the pollutant molecules. Nevertheless, the effect of temperature on NMs has special characteristics different from traditional adsorbents. NPs are prone to agglomeration at high temperatures, which is due to the Ostwald ripening process driven by their high surface energy [86,87]. Small-sized NPs dissolve first due to their high surface energy, and then deposit on the surface of larger particles, resulting in an increase in particle size and a decrease in specific surface area [86,87]. Regarding photochemical reaction, temperature influences both the reaction rate and efficiency of the formation of free radicals. The concentration of NPs governs the number of active sites and the total adsorption capacity. While using greater amounts of NPs enhances pollutant elimination normally due to the higher number of accessible active sites, too high doses can result in aggregation of particles and reduce the efficiency of adsorbent utilization. Thus, optimal doses have been established in past researches using CNTs in the cleanup of pollutants. The result of the Pb(II) removal process using GO-Gd_2_O_3_ has shown that maximum efficiency is reached with a specific dose [77]. In practical applications, it is necessary to determine the cost-effective dosage of NMs through experiments. The initial concentration of pollutants affects the adsorption driving force and the saturation of adsorption sites. The abundance of active sites on NMs on their own is not enough to ensure the reliable removal of pollutants in conditions with low concentration. Further, as we increase the concentration of the pollutant substance, the force driving the adsorption process substantially increases, which in turn increases the adsorption capacity. However, after the concentration reaches a certain point, active sites are saturated and the removal rate drops. In the past, the removal efficiency of pollutants using carbon-based NPs (CNPs) has been measured at levels from 75% to 98%, with higher levels of efficiency typical for smaller initial concentrations of the pollutant [80,81,82]. The metals’ presence such as Pb(II) and Cd(II) indicates that the initial concentration might affect types of adsorption isotherms and adsorption capacity [65]. In real wastewater, there are many ions and organic substances, e.g., dissolved Na^+^ ions, Ca^2+^ ions, Mg^2+^ ions, Cl^−^ ions, NO_3_-ions, and SO_4_^2−^, which may compete with pollutant ions for adsorption or interact with NMs. For example, recent studies have shown that ZIF-8-EGCG has a selective order of Cu^2+^ >> Cr^+6^ > Cd^2+^ > Ni^2+^ [67,68]. The adsorption performance of MXene material in a complex aqueous matrix was significantly affected by coexisting ions, and the selectivity needed to be enhanced by a surface functionalization strategy. Humic acid and other natural organic substances can coat the surface of NMs, shield the active sites, and reduce the adsorption efficiency. Therefore, the matrix effect must be considered when evaluating the performance of NMs in the actual treatment of wastewater. Contact time influences not only the attainment of adsorption balance, but also an excessive reaction time may give rise to Ostwald ripening which results in an upstairs in the number of nanoparticles [70,71]. NMs have a high number of adsorptions and usually achieve equilibrium within 30 min to 2 h. According to some reports, the biomimetic nacreous cellulose of mollusks, for instance, is capable of attaining equilibrium following the adsorption of Nd^3+^ ions in just 5 min. Other authors state that ZIF-8 and Zeolitic Imidazolate Framework-67 (ZIF-67) are quick adsorbents of Pb^2+^ and Cu^2+^, and that iron nitride oxide carbon nanofiber has a short degradation time of emerging organic contaminants (EOCs). Due to their rapid adsorption kinetics, NMs can be easily used in continuous flow wastewater treatment installations. It is important to consider different factors including initial concentration, adsorbent amount, temperature, and others while deciding on the treatment time. TiO_2_ mainly produces hydroxyl radicals under ultraviolet light (λ < 400 nm), and the optical response range can be extended to the visible region by defect engineering and element doping [70,71,72,73,74,75]. The efficiency of antimicrobial composite was exposed to a dual wavelength (635+808 nm) and it showed to be greater than 99.25%, especially in the case of *Escherichia coli* [78]. Raising light intensity leads to the generation of more electron-hole pairs and thus facilitates photocatalytic activity. However, excessive light intensity can promote electron-hole recombination and photocorrosion even at high catalytic efficiency.

The vast majority (>90%) of published studies have been conducted under idealized laboratory conditions, using single solute or simple multi-solute synthetic wastewater. In a single solute system, almost all NMs exhibit excellent performance (>90% removal rate). However, once competitive ions or coexisting organic compounds are introduced, the removal efficiency generally decreases by 10–30%. For example, the adsorption capacity of GO for Pb^2+^ could be reduced by more than 50% in the presence of competing ions such as Cu^2+^ [65,71,75]. This indicates that the adsorption capacity and other parameters obtained based on single solute systems have limited reference value for practical applications. When the matrix shifts from the synthetic system to the actual wastewater, the performance of NMs can experience a “cliff like” decline. In actual municipal or industrial wastewater, the removal efficiency of NMs is usually 20–40% lower than in synthetic systems. For example, TiO_2_ photocatalytic degradation of antibiotics could reach over 95% in deionized water, but in actual secondary effluent, the efficiency may drop below 60% due to the quenching effect of dissolved organic matter (DOM) and light shielding effect [66,67]. This is the most serious knowledge gap in current research, namely the lack of systematic evaluation of the long-term performance, stability, and anti-interference ability of NMs under actual water conditions. When dealing with industrial wastewater, NMs face more severe challenges such as extreme pH, high salinity, and high concentration of composite pollutants. Under these conditions, the aggregation and surface passivation of NMs intensify, resulting in a much lower number of cycles compared to their performance in municipal wastewater treatment. Furthermore, high material consumption and frequent regeneration costs significantly reduce the technical and economic feasibility.

Although NMs have demonstrated excellent pollutant removal capabilities in laboratory batch experiments, their engineering applications ultimately depend on their ability to effectively integrate with existing wastewater treatment process units such as bioreactors, physical filtration, membrane separation, and advanced oxidation, and maintain stable performance under continuous flow and long-term operating conditions. Existing research has preliminarily explored the application modes of NMs in different process configurations [65,66,67,68,69,70,71,72,73,74,75], but compared to traditional materials, the changes they bring are not always positive, and a comprehensive evaluation is needed from multiple dimensions such as processing efficiency, operational stability, maintenance costs, and secondary risks. Biological treatment (activated sludge process, biofilm process, anaerobic digestion) is the core unit of urban sewage treatment. NMs can be introduced in two ways. One is to directly add them to the bioreactor and use their adsorption or catalytic functions to assist in degradation [90,91]. The other is to use them as a modified layer for fillers or membrane components to improve biological adhesion or filtration performance [90,91]. For example, adding nano-zero-valent iron (nZVI) to an anaerobic digestion tank can promote the conversion of volatile fatty acids in the hydrolysis and acidification stage, and increase methane yield by 20% to 40% [90]. However, the rapid oxidation and aggregation of nZVI resulted in its activity only being maintained for a few days in a continuous flow reactor, far below the expected lifespan in laboratory batch experiments [92]. Compared with traditional iron salt coagulants, although they have no catalytic function, they are simple to add and cost-effective, and have more economic advantages in long-term operation [92]. Advanced oxidation processes (AOPs) such as photocatalytic oxidation, Fenton-like oxidation, and persulfate activation are the core areas in which Nms exhibit catalytic degradation capabilities. In practical engineering, nanocatalysts usually participate in reactions in the form of suspended slurry or a fixed bed. The suspension system has high mass transfer efficiency, but there is a problem of difficult catalyst recovery. Although the fixed bed form (such as coating TiO_2_ on glass beads or ceramic films) is convenient for recycling, the effective illumination area and accessibility of active sites are greatly reduced, resulting in a decrease of 50% to 70% in the apparent reaction rate constant [63,66]. Taking the photocatalytic reactor as an example, in the laboratory, the degradation rate of tetracycline by powder TiO_2_ under simulated sunlight can reach 99%. However, in the pilot-scale flat plate reactor, due to limited light penetration depth and suspended solids shielding in water, the actual effluent removal rate is often lower than 60% [67].

## 5. Technical–Economic Feasibility and Lifecycle Assessment

NMs have demonstrated excellent removal performance in the field of advanced wastewater treatment, but whether a technology can move from the laboratory to engineering applications ultimately depends on its technical–economic feasibility as well as environmental sustainability. Previous research on NMs has mostly focused on removal efficiency and mechanism elucidation, while systematic evaluation of economic costs and environmental impacts throughout the entire lifecycle is relatively weak [92,93]. In fact, even if NMs exhibit a removal rate close to 100% under laboratory conditions, their engineering application prospects are limited if their synthesis costs are high, regeneration is difficult, or there are potential ecological risks. In recent years, more and more research has begun to pay attention to the technical–economic analysis (TEA) and lifecycle assessment (LCA) of treating wastewater by NMs, providing data support for rational evaluation of the feasibility and optimization direction of this technology [92,93,94,95,96,97,98].

**Figure 6:**
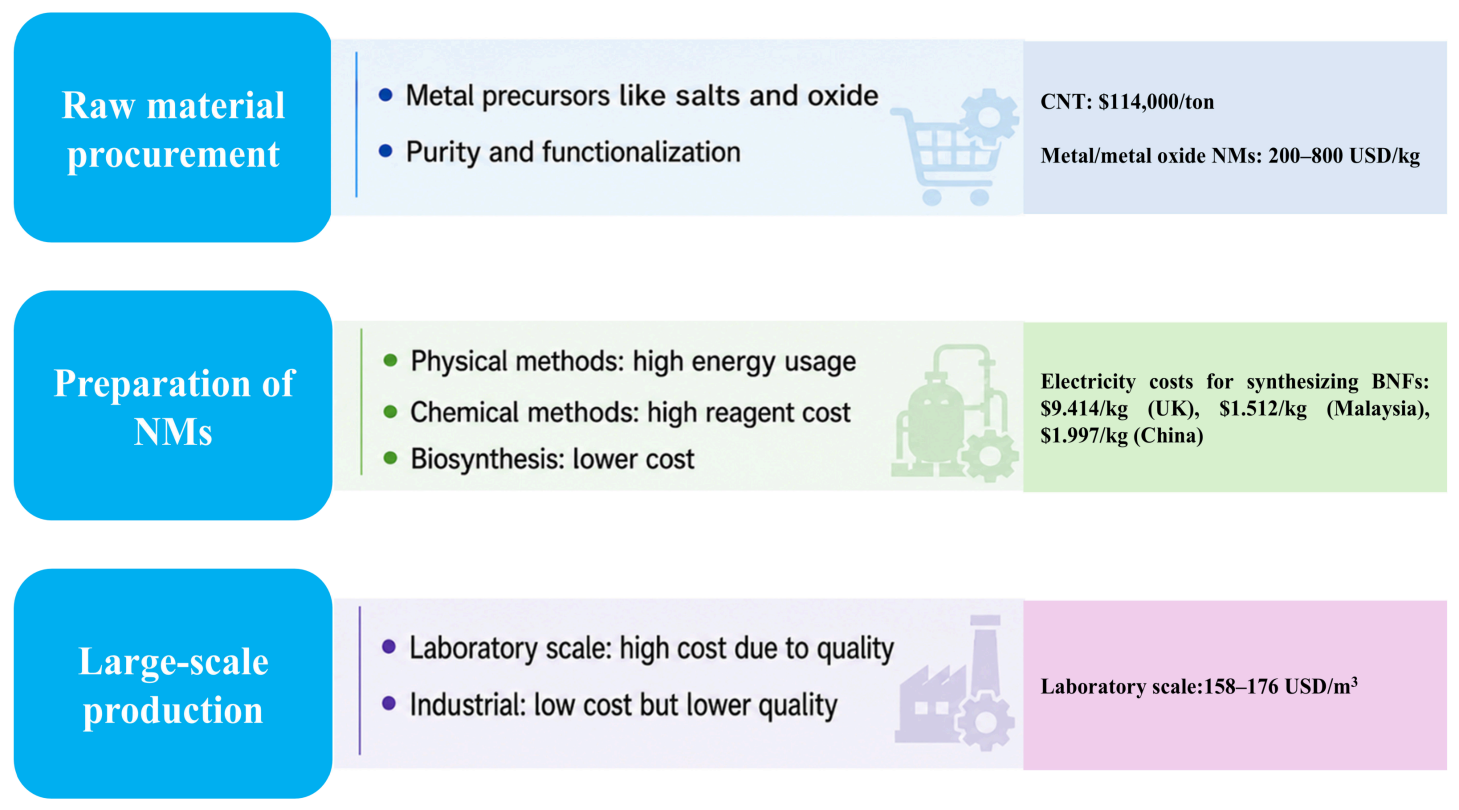
Different cost allocations of NMs in the treatment process of wastewater.

The cost composition of NMs used in the treatment of sewage is a key factor that needs to be comprehensively evaluated. Its economic feasibility directly determines the possibility of transforming from laboratory research to engineering scale. In general, the cost of NMs mainly comprises three components: raw material procurement, preparation of NMs, and large-scale production (Figure 6). Among them, the source and nature of raw materials are basic expenditures. The production cost of carbon-based NMs such as CNTs is extremely high, about USD 114,000/ton, far higher than the USD 1500–2000/ton cost of activated carbon [92,93] (Table 2). The precursors of metal oxide NMs, such as titanium salts, iron salts, zinc salts, etc., have high prices and wide sources (Table 2). The production costs are significantly affected by market supply and demand fluctuations. The green synthesis route utilizes plant extracts, microbial fermentation broth, or agricultural waste as reducing and capping agents, which can significantly reduce raw material costs. For example, the laboratory cost of synthesizing magnetic NPs using plant leaf extracts can be as low as USD 1.26 per kilogram [92,93].

The cost of synthesis and preparation often dominates, especially the energy consumption expenditure. The cost of large-scale production involves the consumption of site, equipment, labor, water, electricity, gas, and other utilities. The relevant research clearly pointed out that in the total production cost of NMs (lanthanum hydroxide/polyvinyl alcohol/polyethyleneimine gained via sol–gel method), the energy cost could be as high as 91% (under the conditions of small-scale synthesis in the laboratory, calculated at a local industrial electricity price of approximately USD 0.10 per kilowatt hour), which was particularly prominent in the preparation process of NMs, because many synthesis methods (such as chemical vapor deposition, laser ablation, arc discharge, high-temperature calcination, etc.) needed to operate under high-temperature, high-pressure, or vacuum conditions [92,93]. It should be emphasized that this proportion reflects the situation at the laboratory scale and is based on the electricity price level in a specific region [92,93]. When the production scale expands to pilot or industrial level, or switches to more energy-efficient synthesis routes (such as room temperature co-precipitation or green biosynthesis), or in regions with lower electricity prices (such as China and Malaysia), the proportion of energy costs can be significantly reduced to 30% to 50% or even lower [92,93]. Taking the black defect type of TiO_2_ NMs as an example, there are differences in the synthesis energy consumption of different morphologies. The synthesis energy consumptions of nanoflowers (BNFs), nanonails (BNNs) and nanowires (BNWs) are 28.607 kWh/kg, 24.945 kWh/kg and 24.364 kWh/kg, respectively [92,93]. It is worth noting that energy costs vary by country and region. The electricity cost for synthesizing BNFs in the UK is approximately USD 9.414 per kilogram, while in Malaysia it is only USD 1.512 per kilogram, and in China it is approximately USD 1.997 per kilogram [94,95,99]. This indicates that the economic feasibility of NM preparation is highly dependent on local energy prices and energy structures. Additionally, reducing agents, stabilizers, templates and solvents constitute chemical costs that cannot be ignored (Figure 6). In contrast, although physical methods (such as ball milling and laser ablation) may avoid the large use of chemical reagents, their equipment investment and power consumption are still high (Figure 6). It is noteworthy that in order to reduce the overall cost, researchers are actively exploring a variety of alternative strategies. Biosynthesis uses renewable biological resources such as plant extracts, microorganisms and natural polysaccharides as reductants and capping agents, which can complete the preparation of NMs under normal temperature and pressure, greatly reducing energy consumption and the use of toxic chemical reagents, thus significantly reducing the cost of synthesis (Figure 6). For example, using agricultural waste extract or microbial culture supernatant to synthesize metal or metal oxide NPs is not only inexpensive and readily available, but also environmentally friendly. At the same time, the preparation of carbon-based NMs from plants or waste biomass (such as carbon quantum dots and porous carbon from biochar or waste fruit shells) is also considered a low-cost alternative route [92,93].

Apart from selecting cheap raw materials and eco-friendly synthesis procedures, the recyclability and reusability of NMs also play an important role that determines the total lifecycle cost [94,95,96,99]. It has been established through earlier studies that in the case where the NMs can be recovered through simple processes such as magnetic separation, centrifuges, or filtration, while keeping their high adsorption capacity, then the unit cost can be drastically decreased [94,95,96,99]. For example, magnetic iron oxide NPs, due to their superparamagnetism, can be quickly separated from water under an external magnetic field, making them easy to recycle and reuse, which compensates for their initial preparation cost [97,98,100]. Conversely, if NMs are difficult to recycle, frequent replenishment of fresh materials is required, resulting in high long-term operating costs [72,97,98,100].

The processing scale has a significant nonlinear impact on the cost of treating wastewater by NMs [101,102,103,104,105,106,107,108]. Laboratory small-scale synthesis often has higher unit costs, while with the expansion of production scale, the cost per unit mass of NMs usually decreases significantly through equipment utilization, batch purchase discounts, and process optimization [109,110]. However, scalability also faces challenges, such as controlling reaction uniformity, ensuring product batch stability, and engineering scaling difficulties in post-processing (washing, drying, dispersion), which indirectly increase practical application costs.

Comparing the cost of NM-based technology with conventional technologies for treating wastewater is a key step in evaluating its engineering application prospects. Table 2 compares NMs with conventional adsorption/oxidation materials from multiple dimensions. In technical–economic analysis, it is not only necessary to compare the unit cost per mass of NMs, but also to comprehensively evaluate the “unit volume wastewater treatment cost” based on their removal rate, selectivity, operating conditions (pH, temperature, coexisting ion effects), and service life for specific pollutants. For instance, although some high-performance CNTs are expensive in unit price (Table 2), their removal rate for heavy metals or organic compounds can reach several times or even tens of times that of traditional activated carbon, which may have better economic efficiency in treating high-concentration or high-risk pollutants [111,112,113]. Similarly, although metal oxide NMs such as zinc oxide and titanium dioxide are relatively mature in synthesis, they require additional ultraviolet or visible light sources during photocatalytic degradation, and this energy consumption must also be included in operating costs [111,112,113]. In addition, magnetic NMs (such as Fe_3_O_4_) can be quickly separated and recovered from water under external magnetic fields due to their superparamagnetism. Taking the bio-nano-composite material guar gum/maghemite (GG/γ-Fe_2_O_3_) as an example, the cost of treating 1000 L of wastewater on a laboratory scale was 158–176 US dollars. However, after four regeneration cycles, the effective treatment cost could be reduced by 70–80%, to 31.6–52.8 US dollars per 1000 L. This discovery emphasizes the core position of recyclability and recycling in the full lifecycle cost of NMs [111,112,113].

**Table 2:** Comparison of cost and performance between NMs and conventional materials.

Comparison Item	Activated Carbon	CNT	Metal/Metal Oxide NMs	Reference
Production cost	1500–2000 USD/ton	Approximately 114,000 USD/ton	200–800 USD/kg	[92,93,100]
Specific surface area	500–1500 m^2^/g	≥1000 m^2^/g	50–900 m^2^/g	
Adsorption/catalytic efficiency	Moderate	High	High	
Recycling	Limited, efficiency degradation	Cycled multiple times partially	Magnetically separated and recycled (magnetic materials)	
Environmental risk	Low	Nanotoxicity risk	Risk of metal ion leaching	
Technology readiness level	Mature industrialization	From laboratory to pilot scale	From laboratory to pilot scale	

At present, most research on the treatment of wastewater with NMs is still in the laboratory batch experiment stage, using simulated wastewater or single pollutant systems, lacking a systematic evaluation of the long-term operational stability, anti-interference ability, membrane fouling or adsorbent blockage of NMs in complex matrices (containing multiple coexisting ions, natural organic matter, suspended particles, etc.) of actual industrial wastewater or urban sewage. The performance degradation under these actual operating conditions will significantly increase the operating and maintenance costs as well as the frequency of material replacement.

LCA is an important tool for comprehensively evaluating the environmental sustainability of NMs for treating wastewater. LCA covers the entire lifecycle stages of raw material acquisition, material synthesis, application operations, and waste disposal [100]. Previous studies have shown that the most significant environmental impacts of NMs throughout their entire lifecycle often occur during the production phase, rather than accidental releases during use [100]. Taking ZnO@Mg(OH)_2_ core-shell nanocomposites as an example, their LCA boundaries covered input terms such as energy input, catalyst materials, textile wastewater, and chemicals, as well as output terms such as treated water and waste catalysts [100,101,102,103]. The evaluation results showed that if the photocatalyst was not disposed of properly, it could have a significant negative impact on the midpoint category of “terrestrial ecological toxicity potential” and the endpoint environmental impact standard of “natural resources” [100,101,102,103]. This suggests that the end of pipe disposal strategy of NMs should be taken seriously as an important component of LCA. In terms of carbon emissions, the study of graded black defect TiO_2_ NMs provides more detailed quantitative data. During the synthesis stage, the carbon emissions of BNFs produced in China were approximately 26.673 kg CO_2_-eq/kg, while in the UK they are only 5.906 kg CO_2_-eq/kg (a difference of about four times) [99]. In the application stage, when treating phenol-containing wastewater (<100 mg/L), the power consumption of BNFs was about 3.99 kWh/m^3^, and the corresponding carbon emissions varied depending on the regional power structure [99]. Malaysia is 2.57 kg CO_2_-eq/m^3^, China is 3.74 kg CO_2_-eq/m^3^, and the UK is 0.89 kg CO_2_-eq/m^3^ [99]. This result indicates that the environmental footprint of NMs is highly dependent on the energy structure of their production and application sites. In areas with a high proportion of fossil fuels, NM technology may bring significant indirect carbon emissions that cannot be ignored. In addition, the environmental fate and ecological risks of NMs after treating wastewater are worthy of attention. NPs may be released into the receiving water body through treated effluent or enriched in residual sludge. Metal-based NMs may undergo metal ion leaching under acidic or oxidative conditions, resulting in toxic effects on aquatic organisms. Although NMs (e.g., Ag, CuO, and ZnO) have excellent antibacterial properties, their continuously released metal ions may have inhibitory effects on functional microorganisms in wastewater biological treatment systems [100,101,102,103]. It implies that the combination of ecological toxicity evaluation with lifecycle assessment (LCA) is required to conduct a thorough assessment of the environmental impact of NMs throughout their entire lifecycle.

## 6. Future Perspectives

NMs have shown great potential in the field of advanced wastewater treatment, and the current studies have also preliminarily verified their excellent performance in adsorption, catalysis, antibacterial and resource conversion. However, there are still many fundamental challenges from laboratory to practical engineering application. In the future, the development direction of NMs should focus on the core goal of “high performance, low risk, easy recovery and large-scale”, systematically break through the current technical bottleneck, and promote its stable operation and long-term application under complex conditions of wastewater.

At present, although there are various methods for preparing NMs. The trade-off between high performance and high cost, along with the difficulty of balancing simplicity and controllability, persists. In the future, more attention should be paid to the “rational design” of nanocomposites, that is, starting from the molecular structure of pollutants, deducing the required surface chemical properties, pore structure, active site types, and spatial distribution of NMs. For example, for specific heavy metal ions or antibiotic molecules, the optimal ligand functional group type and spatial configuration can be predicted through theoretical calculations and molecular simulations to guide material synthesis. In terms of preparation methods, green synthesis routes will become an important development direction. Currently, biomass resources such as plant extracts, microbial fermentation broths, and natural polysaccharides have been preliminarily used for the reduction and stabilization of NMs, but the accuracy of morphology and particle size control is still far lower than that of chemical methods. In the future, a combination of metabolic engineering and synthetic biology methods should be used to selectively modify microorganisms, enabling them to stably produce specific reductases or functional peptides and achieve precise regulation of NP nucleation and growth processes.

The composition of real wastewater is extremely complex, with various coexisting ions, natural organic matter, surfactants, microorganisms, and their metabolites. The interactions between these components and NMs are often overlooked in laboratory studies under simplified conditions. Future research papers should clarify the matrix category to which their research belongs and discuss the comparability of their results with existing studies in that category, as well as the limitations of extrapolating to more complex systems. Funding agencies and journals should encourage confirmatory research in real water bodies or at least complex synthetic matrices. In the future, it is necessary to systematically study the “service behavior” of NMs in complex matrices, including their dispersion state, surface chemical evolution, active site shielding, dissolution toxicity changes, etc. Of particular concern is that NMs may undergo “dynamic surface transformation” in actual water bodies. For instance, environmental components such as sulfides, phosphates, and humic acids can form a coating layer on the surface of NMs, altering their original adsorption or catalytic properties. This “aging effect” can both inhibit the activity of NMs and unexpectedly enhance their selectivity towards specific pollutants. Therefore, in the future, in situ characterization techniques should be developed, such as liquid phase transmission electron microscopy, in situ Raman spectroscopy, synchrotron radiation X-ray absorption spectroscopy, etc., to track the morphology and chemical state evolution of NMs in real wastewater. At the same time, an “environmental tolerance evaluation index system” for NMs should be established, including the ability to resist ion interference, organic pollution, and biological pollution. It can be used as a prerequisite for material screening. Reliable prediction models can be provided for engineering applications of NMs only by comprehensively understanding the evolution laws of NMs under complex conditions of wastewater.

At present, the understanding of the interaction between NMs and microbial aggregates such as activated sludge and biofilm is mostly at the phenomenological level. In the future, the following key scientific issues are worth studying. Firstly, the binding selectivity of NMs with different functional groups (such as carboxyl, phosphate, thiol, amino, etc.) in EPS and their influence on the spatial conformation of EPS need to be clearly defined. Secondly, how the physicochemical property changes induced by NMs regulate the migration, transformation, and toxic release of NMs conversely should be answered. Lastly, the interference mechanism of NMs on microbial community sensing systems and their impact on biofilm formation, stability, and detachment behavior ought to be explained. On this basis, the strategy of “actively regulating EPS” can be explored. For example, by adding signal molecules, changing hydraulic shear force, or adjusting carbon nitrogen ratio, microorganisms can be induced to secrete more EPS with protective or adsorption functions, thereby weakening the biological toxicity of NMs and enhancing the overall system’s tolerance to NMs. This approach is essentially a form of “biological adaptive management” and is expected to become an important auxiliary means for the safe application of NMs. In addition, there is a lack of systematic research on the effects of NMs on the expression levels of microbial functional genes such as amoA, nirS, nosZ, etc. In the future, macro transcriptomics and single-cell analysis techniques should be combined to reveal the disturbance mechanism of NMs on nitrogen and phosphorus metabolism networks at the gene expression level, providing molecular level theoretical support for regulating the stable operation of sewage treatment systems.

At present, the majority of research regards NMs as “disposable consumables” and ignores their recycling and regeneration issues after wastewater treatment. In the future, core-shell structured magnetic NMs (such as Fe_3_O_4_@SiO_2_) should be developed to enhance their stability under harsh conditions, as well as stimulus responsive intelligent NMs (temperature/pH/photoresponsive) to achieve reversible dispersion and aggregation, and simplify the recycling process. At the same time, it is necessary to establish a standardized “performance reversibility evaluation method”, which takes the activity retention rate, structural integrity, and metal leaching rate after multiple cycles as the core parameters for material engineering applications.

Currently, vast research on NMs still focuses on simulating wastewater or single-pollutant systems, lacking long-term operational validation in actual wastewater. It is important to facilitate the translation of laboratory research to industrial-scale applications, selecting typical industrial wastewater (such as printing and dyeing, pharmaceuticals, electroplating, papermaking, etc.) and secondary effluent from urban sewage treatment plants as research objects, and conducting pilot-scale studies in continuous flow reactors (such as fixed bed, fluidized bed, membrane reactors, etc.). These studies should focus on the following aspects: the long-term stability and anti-pollution ability of NMs in actual wastewater; its compatibility with existing processing units such as biological tanks, sedimentation tanks, and disinfection tanks; and the optimal addition point and method in the entire sewage treatment process. At the same time, the possible side effects of nano materials such as membrane pollution, foam generation, and sludge property change should be evaluated. Technical–economic analysis should start from the perspective of the lifecycle, comprehensively accounting for the energy consumption, material consumption, and environmental emissions of the production, transportation, addition, recycling, regeneration, and final disposal stages of NMs. It is particularly important to note that the “unit volume treatment cost” of different NMs under the same water quality conditions should be used as the core evaluation indicator. In addition, a cost–benefit database for treating wastewater with NMs should be established, covering typical cases of different material types, treatment scales, and water quality conditions, to provide data support for engineering decision-making.

While promoting the application of NMs, it is necessary to address their potential environmental and health risks. NMs may enter water bodies, soils, and organisms through effluent discharge, sludge disposal, and other pathways during wastewater treatment. In the future, it is necessary to systematically study their fate behavior in different environmental media, especially the “long-term low-dose exposure” effect. We should develop traceability technologies such as element fingerprinting and isotope tracing, establish long-term exposure models based on environmental concentrations, and evaluate their chronic and intergenerational toxicity to aquatic organisms, soil microorganisms, and ecosystems. Standardization construction is the inevitable path for the engineering application of NMs. At present, the International Organization for Standardization (ISO) and American Society for Testing and Materials (ASTM International) have developed multiple relevant standards [114,115]. In the future, we should learn from and participate in the formulation and revision of such standards, accelerate the establishment of a standard system covering performance testing, ecological toxicity assessment, and technical and economic analysis, and provide a scientific basis for the safe and standardized application of NMs.

The research on NMs in wastewater treatment is essentially a cutting-edge field that intersects multiple disciplines such as materials science, environmental engineering, molecular biology, and computational chemistry. In the future, interdisciplinary collaboration should be strengthened to promote the full chain research mode of “theoretical simulation material preparation performance evaluation mechanism analysis engineering verification”. It is particularly worth emphasizing that standardization construction is the only way for NMs to move towards engineering applications. Currently, there is a lack of unified standards for performance evaluation methods, toxicity testing procedures, recovery rate calculation methods, and lifecycle assessment frameworks for NMs, which makes it difficult to compare different studies horizontally. In the future, we should actively promote the development of industry standards, group standards, and even national standards, covering key aspects such as naming, characterization methods, performance testing, ecological toxicity evaluation, and technical–economic analysis of NMs.

## 7. Conclusions

The progress made in NM research in deep wastewater treatment is systematically analyzed in this paper. The individual components of the review include preparation techniques, cleaning methods, efficacy, costing, and technical problems. Overall, preparation methods can be grouped into two types roughly: top-down and bottom-up methods. The physical method (top-down) and the chemical method (bottom-up) each have their own advantages and disadvantages. While the physical method is easier to implement, it has limitations in particle size control; conversely, the chemical method has great accuracy but it is expensive and leads to low yield. Green synthesis is environmentally friendly and economical, but the control of morphology and particle size still needs to be improved. Future research must go beyond simple binary analysis or listing, establish a quantitative relationship of “preparation structure performance cost”, and make rational choices and designs based on specific treatment goals and conditions (such as pollutant types and treatment scales). The selection and matching of carriers are equally crucial, requiring a shift from empirical trial and error to system theory research based on interface interactions. To solve the problem of the easy agglomeration and difficult recovery of nano powder, the nano powder was loaded on a millimeter-scale porous carrier to construct a composite, and the particles were grown in situ by impregnation, precipitation and other methods. The particle size was controlled at 2–50 nm by the restriction of the carrier pore, and the stability of the load was determined by the interface effect. The removal mechanism includes adsorption, catalysis, ion exchange, antibacterial, etc. These mechanisms can be systematically classified into physical separation, chemical transformation, and complete destruction. In terms of engineering application prospects, magnetic core-shell composite materials (such as Fe_3_O_4_@SiO_2_ functionalized materials) and single atom catalysts (such as Fe-N-C) are considered to have the greatest potential. The former solves the recycling problem, while the latter has significant advantages in catalytic activity and atomic utilization. The vast majority of research is conducted in highly idealized single solute synthesis systems, while their performance in multi-solute systems and real wastewater decreases significantly. The main bottlenecks for achieving large-scale applications are: (1) under real and complex water quality conditions, the performance of NMs usually decreases by 30–70% compared to ideal systems; (2) the lack of long-term operational stability and anti-pollution capability; and (3) the lack of a unified standardized evaluation system and full lifecycle risk data. Based on current progress, it is expected that NMs can achieve demonstrative engineering applications in the deep treatment of some industrial wastewater (such as electroplating wastewater, printing and dyeing wastewater) and municipal wastewater upgrading and transformation in the next 5–10 years. However, their popularization in large-scale municipal wastewater treatment still faces significant challenges. The performance data of NMs indicate that they can achieve a removal rate of over 99% for specific pollutants, but their effectiveness in practical engineering (usually under optimal conditions of 70–90%) is constrained by multiple factors. In the future, it is urgent to conduct in-depth research on green controllable synthesis, complex matrix service behavior, microbial interaction mechanisms, efficient recycling and regeneration, and full lifecycle risk assessment. It is favorable to promote the sustainable and large-scale application of NMs in the field of sewage treatment through interdisciplinary cooperation and standardization construction.

## Figures and Tables

**Table 1:** Preparation methods of NMs.

Category	Method	Mechanism/Description	Advantage	Disadvantage	Applicability of Treating Wastewater	Key Limitation and Challenge	Reference
Physical methods	Laser ablation	High-energy laser vaporizes materials to form NPs in gas/liquid	High purity, medium versatility	Expensive, low yield	Low	Difficult to scale up the production of materials required for treating wastewater due to high costs	[16]
	Ball milling	Mechanical grinding of bulk materials into NPs	Simple, scalable, solvent-free	Poor control of size and shape, possible pollution	Moderate	Wide particle size distribution, introduction of impurities may lead to a decrease in catalytic activity	[18]
	Sputtering	Eject atoms from a target by ion bombardment	Precise control and high purity	Long deposition time, complex equipment	Low	High costs of equipment and operating, low yield	[19]
	Arc discharge	The arc between the electrodes vaporizes the material	Simple and low cost for carbon nanostructures	High temperature, harmful smoke	Low	High energy consumption in high-temperature processes, product purity affected by electrode materials	[21]
	Plasma synthesis	Formation of NPs by gasification of materials with plasma energy	Fast, clean, industrializable scale	Expensive equipment	Low	High costs of equipment investment and operation	[17]
	Lithography	Precise preparation of NPs by etching or embossing the surface	The desired shape and structure obtained for microelectronics and integrated circuits	Expensive, complex equipment	Extremely low	Extremely expensive equipment, complex process, extremely low yield	[22]
	Electron beam evaporation	The material heated by electron beam in vacuum to evaporate	Materials with high purity	Only for materials with heat resistance	Extremely low	Only applicable to heat-resistant materials, with high equipment investment and operating costs	[23]
	Thermal evaporation	Deposition by high-temperature vacuum evaporation	Environment friendly, no by-product emission	Limited control of deposition, narrow application scope	Extremely low	Limited control over sedimentation processes and narrow applicability	[19]
	Gas phase condensation	Metal vapor cooling to form NPs	Controllable phase, good crystallinity and narrow particle size distribution	Relatively low yield	Moderate	Relatively low yield	[31]
	Inert gas condensation	Metal vapor cools in inert gas to form NPs	Simple and clean	Low yield, vacuum required	Low	Very low yield	[32]
Chemical methods	Vapor deposition	Deposition of gaseous precursors to form films or particles	Good uniformity, controllable surface morphology, large scale	Chemical hazards, need for vacuum and high temperature	Moderate	Complex process control and high temperature requirements result in extremely high overall costs	[17]
	Hydrothermal/solvothermal synthesis	Crystallization under high temperature and pressure in sealed autoclave	Crystalline particles, good morphology control	High pressure equipment and high temperature	High	High voltage operation poses safety risks and limits mass production capacity	[19]
	Sol–gel synthesis	The precursor hydrolyzed to form gel and dried to obtain NPs	Good control of composition, low operating temperature	Sensitive to moisture, time consuming, shrinkage problems	High	Time-consuming, shrinkage issues during scaling leads to cracking affecting material integrity	[19]
	Co-precipitation	Simultaneous precipitation of components in solution	Simple, cost-effective	Poor control of particle size distribution, time-consuming	High	Uneven particle size distribution and poor repeatability between batches	[19]
	Template-assisted Synthesis	Growth of NPs in templates	High control of shape/size	Formwork removal and cost	High	Complex and expensive for the template removal process	[24]
	Microemulsion	Formation of NPs in confined micelles	Narrow particle size distribution	Low yield, need to remove surfactants	Moderate	The extensive use of organic solvents and surfactants brings environmental and economic burdens	[24]
	Chemical reduction	Reduction of metal ions to NPs using reducing agents	Simple, widely used in metal	Toxic reductant	High	Difficult post-processing of toxic reducing agents	[28]
	Electrochemical synthesis	Reduced metal ions on the electrode surface	Size controlled by voltage/current	Only for conductive materials	Moderate	Limited scale-up by electrode area and mass transfer	[24]
	Reverse micellar	Reactants confined in surfactant stabilized nanodroplets	Precise dimension	Difficult to scale up, need to remove surfactants	Moderate	Low yield, difficult to scale up	[20]
	Spray pyrolysis	The atomized precursor fluid drops thermally decomposed to form particles	Suitable for mass production	High temperature required	High	Requiring high-temperature environment, high energy consumption	[26]
	Polymerization	Formation of NPs during polymerization	Suitable for drug delivery and coating, a wide range of applications	Involving stabilizer and surfactant	Moderate	The stabilizers and surfactants used pose environmental risks	[27]
	Flame spray pyrolysis	Combustion of metal precursor in flame	Large scale, fast and strong industrial applicability	Particle agglomeration, temperature sensitive	High	High-temperature process leads to severe particle agglomeration	[26]
	Sonochemical synthesis	Ultrasonic-induced cavitation and particle formation	Room temperature operation, fast nucleation	Limited control of dimensional	High	Limited control over size and morphology	[29]
	Photochemical synthesis	Light-induced reduction/oxidation of metal ions	No need for high temperature	Need UV light source, slow reaction rate	Moderate	Slow reaction rate	[30]
	Supercritical fluid synthesis	Formation of NPs using supercritical CO_2_ or water as solvent	Clean with free residue	High voltage system required	Moderate	High costs of equipment, harsh operating conditions	[32]
	Molecular self-assembly	Molecules or atoms spontaneously arranged into ordered nanostructures	No external template required	Limited material types	Moderate	Limited types of applicable materials	[32]
	Microwave-assisted combustion	Combustion synthesis promoted by microwave heating	Fast and energy saving	Precise control required to avoid agglomeration	High	Highly required precise control of reaction conditions	[33]
Biological method	Green synthesis	Use of plant extracts, enzymes, bacteria or fungi	Environmental protection and good biocompatibility	Difficult to control morphology and size, additional synthesis steps	High	Lack of precise control over particle size, morphology and crystal phase resulting in poor batch stability	[25]
	Interfacial polymerization	Formation of NPs at the interface of two immiscible liquids	Adjustable particle size and easy operation	Complex nature of control interface	Moderate	Limitations on synthesizing a large number of free NPs	[34]

## Data Availability

The original contributions presented in this study are included in the article/Supplementary Material. Further inquiries can be directed to the corresponding authors.

## Supplementary Materials

The following supporting information can be downloaded at: https://www.mdpi.com/article/10.3390/molecules31172991/s1, Table S1. Purification of various pollutants by NMs. Refs. [116,117,118,119,120,121,122,123,124,125,126,127,128,129,130,131,132,133,134,135,136,137,138,139,140,141,142,143,144,145,146,147] are cited in the Supplementary Materials.
