# Supplementary material for: Carrier-Assisted Nanomaterials and Microbial Dynamics in Advanced Wastewater Treatment: A Review
Source: Molecules. 2026 Aug 26;31(17):2991. doi: 10.3390/molecules31172991 (PMC13567525; doi:10.3390/molecules31172991)
Supplement: Supplementary file 1 [file molecules-31-02991-s001.zip › molecules-4499115-supplementary.pdf]

Review

Carrier-Assisted Nanomaterials and Microbial Dynamics in Advanced Wastewater Treatment: A Review

Zhongchuang Liu [1,*] , Siu Hua Chang [2], Gilles Mailhot [3,*], Mohsen Taghavijeloudar [4] and Valentin Romanovski [5]

[1] Department of Environmental Engineering Technology, College of Power Engineering, Chongqing Electric Power College, No. 9, Electric Power Fourth Village, Jiulongpo District, Chongqing 400053, China

[2] Waste Management and Resource Recovery (WeResCue) Group, Chemical Engineering Studies, College of Engineering, Universiti Teknologi MARA, Cawangan Pulau Pinang, Permatang Pauh 13500, Penang, Malaysia; shchang@uitm.edu.my

[3] Institut de Chimie de Clermont-Ferrand, Université Clermont Auvergne—CNRS, F-63000 Clermont-Ferrand, France

[4] Department of Civil Engineering, Mazandaran Institute of Technology, Babol 4748161136, Iran; taghavi@mit.ac.ir

[5] Department of Materials Science and Engineering, University of Virginia, Charlottesville, VA 22904, USA; rvd9ar@virginia.edu

* Correspondence: liuzhongchuang@cqepc.edu.cn (Z.L.); gilles.mailhot@uca.fr (G.M.)

Supplementary materials

Table S1. Purification of various pollutants by NMs. 1

NM	Pollutant	Optimum condition	Removal rate (%)	Matrix type	Reference
MWCNTs-KOH@Ni	Pb (II)	pH: 5.5, T: 303 K, 30 min	91.2	S	[1]
	As (V)		88.5		
	Cd (II)		80.6		
rGO hydrogel (rGOH)	Pb (II)	pH: 4, T: 298 K, 5 min	80	S	[2]
	Cd (II)		80		
GO–Gd_2O_3 nanocomposite	Pb (II)	pH: 4, T: 298 K, 1080 min	99	MU	[3]
rGO–Starch composite (rGO/WS)	Cu (II)	pH: 8, 24 min	90.34	S	[4]
CQD-CNTs	Methylene blue	pH: 6, T: 313 K, 5 min	99.5	S	[5]
MWCNTs Cellulose beads	Methylene blue	pH: 7, T: 25 ℃, 1.07 min	97.05	S	[6]
Fe@MWCNTs	Azorubine	pH: 5, T: 24–26 ℃, 30 min	95	S	[7]
MWCNTs	Ismate violet 2R	pH: 4, T: 30 ℃, 120 min	88.2	S	[8]
MWCNTs	Methyl orange	120 min	16	S	[9]
	Rhodamine B		60		
Co-N@CNTs	Oxalic acid	pH: 4.5	92	S	[10]

NM	Pollutant	Optimum condition	Removal rate (%)	Matrix type	Reference
MWCNT–amino acid	Ibuprofen	pH: 4, room temperature	98.4	S	[11]
Co@CoO/NC	Rhodamine	pH: 8, 60 min	98	S	[12]
	Tetracycline		99		
FMWCNTs	Total organic carbon	pH: 7, T: 34 °C, 49 min	93.6	I	[13]
(MWCNTs)-$SiO_{2.5}$ composite	Toluene	T: 30–60 °C, 10–30 min	73	S	[14]
GO	Sodium diclofenac	pH: 6.2, T: 25–45 °C, 300 min	74	S	[15]
	Pb^{2+}	Neutral pH	>90		[16]
CNFs	Alkylphenols	Room temperature, 5 min	73.58–85.76	S	[17]
ZnO NPs	Methyl orange	120 min	60	S	[18]
	Amaranth		78		
ZnO NPs (Ni tubular thin films)	Textile dyes	60 min	91.21	S	[19]
ZnO NPs (hydrogel composite)	*Escherichia coli*	35 min	70	S	[20]
ZnO nanorods	Congo red and methyl orange	75 min	96	M	[21]

NM	Pollutant	Optimum condition	Removal rate (%)	Matrix type	Reference
	Methylene blue	120 min	69-73		[22]
CuO NPs	NO_3^- ions	120 min	66.9-71.56	I	[23]
	Acrylic acid	180 min	75-78		[24]
	Co	20 min	73.2		[25]
	Pb		80.8		
	Ni		72.4		
	Cd		64.4		
	Cr		91.4		
CuO NPs (loaded into nano porous activated carbon)	Methylene blue	180 min	93.51	S	[26]
Ag_2O nano fibers	Methylene blue	150 min	97.57	S	[27]
Ag_2O nano fibers (loaded polyacrylonitrile membrane)	Methylene blue	15 min	99.5	S	[28]
	Pb^{2+}		99.7		
Ag_2O NPs	Methylene blue	20-35 min	88.6-99.5	S	[29,30]
	Methyl orange	20 min	90.2		

NM	Pollutant	Optimum condition	Removal rate (%)	Matrix type	Reference
	Rhodamine B		96.5		
Ag_2O NPs (Ag_2O-Mn-NPs)	Malachite green	60 min	99	S	[31]
TiO_2 NPs	Methylene blue	120 min	85.5-92	S	[32,33]
Doped TiO_2 NPs (Ag surface modification)	DPPH radical	120 min	31-70	S	[34]
Coated TiO_2 NPs (Ag NP coated)	Methylene blue	120 min	90.9	S	[35]
α-Fe_2O_3	COD	8 h	82.8	I	[36]
	TDS		47.6		
	TSS		75.7		
	Yellow RR	6 h	76.6	S	[37]
Fe_2O_3 (CuO-CeO_2-Fe_2O_3 nanocomposite)	Red 60	3 h	77	I	[38]
CMC-Fe_3O_4	Pb^{2+}	12 min	68.4	S	[39]
Ag NPs	Bacteria	pH: 6-8, ambient	>99	S	[40]
	Hg^{2+}, Cd^{2+}	temperature	80		
nZVI	Cr (VI)	Anaerobic, pH: 4-7	>95	S	[40]

NM	Pollutant	Optimum condition	Removal rate (%)	Matrix type	Reference
Nano-zeolites	NH_4^+, Pb^{2+}, bacteria	pH: 6-8	80-90	S	[40]
Nano-clays	Pb^{2+}, Cu^{2+}, dyes	Neutral pH	>90	S	[40]
TiO_2–GO	Dyes, antibiotics	Solar light, pH: 6–8	>95	S	[40]
MXenes	Cr (VI), bacteria	pH: 6–8	>99	S	[40]
Poly(β-CD- BPDA)	Methylene blue	60 min	97	S	[41]
Poly (LIM-co-DVB-co-VIM)	Pb (II)	60 min	99.1	S	[42]
PANI@PCDP	Cr (VI)	20 min	99	M	[43]
	BPA	30 min	88.9		
Ce-UiO-66	Methylene blue	60 min	95	S	[44]
MIL-101(Cr)- NH_2	2,4-dichlorophenoxyacetic acid	5 min	87.1	S	[45]
MnO_2	Pb (II)	30 min	94	S	[46]
PTS-Chol- MSN	Sulfamethoxazole	60 min	50	M	[47]
	Diclofenac	360 min	94		
G3DTF	Hg (II)	120 min	99.7	S	[48]

NM	Pollutant	Optimum condition	Removal rate (%)	Matrix type	Reference
MAA-$ZnCrFeO_4$/HKUST-1	Methylene blue	15 min	70	S	[49]
MWCNTs/PANI/Fe_3O_4	Methylene blue	180 min	87.7	S	[50]
	Congo red		81.8		
Chitosan-TiO_2	Methylene blue	120 min	95	S	[51]

S: single-solute systems; MU: municipal wastewater; I: industrial wastewater; M: multi-solute synthetic systems.

References

1. Egbosiuba, T.C.; Egwunyenga, M.C.; Tijani, J.O.; Mustapha, S.; Abdulkareem, A.S.; Kovo, A.S.; Krikstolaityte, V.; Veksha, A.; Wagner, M.; Lisak, G. Activated multi-walled carbon nanotubes decorated with zero valent nickel nanoparticles for arsenic, cadmium and lead adsorption from wastewater in a batch and continuous flow modes. J. Hazard. Mater. 2022, 423, 126993.
2. Hu, Y.; Gao, H.; Yang, Q.; Zhou, W.; Sun, C. Adsorption of Pb^{2+} and Cd^{2+} on reduced graphene oxide hydrogel prepared from natural cryptocrystalline graphite. Colloids Surf. A Physicochem. Eng. Asp. 2022, 642, 128630.
3. Lee, S.; Lingamdinne, L.P.; Yang, J.-K.; Koduru, J.R.; Chang, Y.-Y.; Naushad, M. Biopolymer mixture-entrapped modified graphene oxide for sustainable treatment of heavy metal contaminated real surface water. J. Water Process Eng. 2022, 46, 102631.
4. Bulin, C.; Zhang, B.; Guo, T.; Ma, Z.; Li, B.; Zhang, Y.; Xing, R.; Ge, X. Graphene oxide–starch composite as an efficient adsorbent for removing Cu(II): Removal performance and adsorption mechanism. Res. Chem. Intermed. 2021, 47, 3825-3852.
5. Chen, Q.; Wang, H.; Tang, X.; Ba, Z.; Zhao, X.; Wang, Y.; Deng, H. One-step synthesis of carbon quantum dot-carbon nanotube composites on waste eggshell-derived catalysts for enhanced adsorption of methylene blue. J. Environ. Chem. Eng. 2021, 9, 106222.
6. Ding, F.; Ren, P.; Wang, G.; Wu, S.; Du, Y.; Zou, X. Hollow cellulose-carbon nanotubes composite beads with aligned porous structure for fast methylene blue adsorption. Int. J. Biol. Macromol. 2021, 182, 750-759.
7. Madihi-Bidgoli, S.; Asadnezhad, S.; Yaghoot-Nezhad, A.; Hassani, A. Azurobine degradation using Fe2O3@multi-walled carbon nanotube activated peroxymonosulfate (PMS) under UVA-LED irradiation: Performance, mechanism and environmental application. J. Environ. Chem. Eng. 2021, 9, 106660.
8. Abualnaja, K.M.; Alprol, A.E.; Ashour, M.; Mansour, A.T. Influencing Multi-Walled Carbon Nanotubes for the Removal of Ismate Violet 2R Dye fromWastewater: Isotherm, Kinetics, and Thermodynamic Studies. Appl. Sci. 2021, 11, 4786.
9. Madhurima, V.P.; Kumari, K.; Jain, P.K. Synthesis and study of carbon nanomaterials through arc discharge technique for efficient adsorption of organic dyes. Diam. Relat. Mater. 2024, 141, 110538.
10. Wang, Y.; Ren, N.; Xi, J.; Liu, Y.; Kong, T.; Chen, C.; Xie, Y.; Duan, X.; Wang, S. Mechanistic Investigations of the Pyridinic N–Co Structures in Co Embedded N-Doped Carbon Nanotubes for Catalytic Ozonation. ACS EST Eng. 2021, 1, 32-45.
11. Hanbali, G.; Jodeh, S.; Hamed, O.; Bol, R.; Khalaf, B.; Qdemat, A.; Samhan, S. Enhanced ibuprofen adsorption and desorption on synthesized functionalized magnetic multiwall carbon nanotubes from aqueous solution. Materials. 2020, 13, 3329.
12. Yang, G.; Li, Y.; Yang, S.; Liao, J.; Cai, X.; Gao, Q.; Fang, Y.; Peng, F.; Zhang, S. Surface oxidized nano-cobalt wrapped by nitrogen-doped carbon nanotubes for efficient purification of organic wastewater. Sep. Purif. Technol. 2021, 259, 118098.
13. Adewoye, T.L.; Ogunleye, O.O.; Abdulkareem, A.S.; Salawudeen, T.O.; Tijani, J.O. Optimization of the adsorption of total organic carbon from produced water using functionalized multi-walled carbon nanotubes. Heliyon. 2021, 7, e05866.
14. Liu, W.; Li, Z.; Zhang, S.-P.; Jian, W.-W.; Ma, D.-Z. Adsorption performance of multi-walled carbon nanotube-SiO_2 adsorbent for toluene. J. Fuel Chem. Technol. 2021, 49, 861-872.
15. Guerra, A.C.S.; de Andrade, M.B.; Tonial dos Santos, T.R.; Bergamasco, R. Adsorption of sodium diclofenac in aqueous medium using graphene oxide nanosheets. Environ. Technol. 2021, 42, 2599-2609.
16. Rahman, M.A.; Hasnain, S.M.M.; Zairov, R.; Kumar, R. Recent advances in nanocomposites and nano-adsorbents for heavy metal and dye removal from water. Clean. Water. 2026, 5, 100215.
17. Zhou, S.; Guo, J.; Zou, Y.; Wang, L.; Kaw, H.Y.; Quinto, M.; Meng, L.-Y.; Dong, M. Fast removal of phenolic compounds from water using hierarchical porous carbon nanofibers membrane. J. Chromatogr. A. 2022, 1685, 463624.
18. Zafar, M.N.; Dar, Q.; Nawaz, F.; Zafar, M.N.; Iqbal, M.; Nazar, M.F. Effective adsorptive removal of azo dyes over spherical ZnO nanoparticles. J. Mater. Res. Technol. 2020, 8, 713-725.
19. Biswal, H.J.; Srivastava, T.; Vundavilli, P.R.; Gupta, A. Facile fabrication of hydrophobic ZnO nanostructured nickel microtubes through pulse electrodeposition as promising photocatalyst for wastewater remediation. J. Manuf. Process. 2022, 75,

538-551.
20. Bwatanglang, I.B.; Mohammad, F.; Janet, J.N.; Dahan, W.M.; Al-Lohedan, H.A.; Soleiman, A.A. Biosorption of Escherichia coli Using ZnO-Trimethyl Chitosan Nanocomposite Hydrogel Formed by the Green Synthesis Route. Gels 2023, 9, 2310-2861.
21. Norbert, A.; Shaji, S.; Philip, R.R. Electrochemically anodized solid and stable ZnO nanorods as an adsorbent/nanophotocatalyst: ROS mediated degradation of azo dyes congo red and methyl orange. J. Clean. Prod. 2023, 428, 139466.
22. El Golli, A.; Fendrich, M.; Bazzanella, N.; Dridi, C.; Miotello, A.; Orlandi, M. Wastewater remediation with ZnO photocatalysts: Green synthesis and solar concentration as an economically and environmentally viable route to application. J. Environ. Manag. 2021, 286, 112226.
23. Rahdar, S.; Pal, K.; Mohammadi, L.; Rahdar, A.; Goharniya, Y.; Samani, S.; Kyzas, G.Z. Response surface methodology for the removal of nitrate ions by adsorption onto copper oxide nanoparticles. J. Mol. Struct. 2021, 1231, 129686.
24. Baylan, N.; ˙Ilalan, ˙I.; ˙Inci, ˙I. Copper oxide nanoparticles as a novel adsorbent for separation of acrylic acid from aqueous solution: Synthesis, characterization, and application. Water Air Soil Pollut. 2020, 231, 465.
25. Eid, A.M.; Fouda, A.; Hassan, S.E.; Hamza, M.F.; Alharbi, N.K.; Elkelish, A.; Alharthi, A.; Salem, W.M. Plant-Based Copper Oxide Nanoparticles; Biosynthesis, Characterization, Antibacterial Activity, Tanning Wastewater Treatment, and Heavy Metals Sorption. Catalysts. 2023, 13, 345-378.
26. Prajapati, A.K.; Mondal, M.K. Comprehensive kinetic and mass transfer modeling for methylene blue dye adsorption onto CuO nanoparticles loaded on nanoporous activated carbon prepared from waste coconut shell. J. Mol. Liq. 2020, 307, 112-149.
27. Kadam, J.; Dhawal, P.; Barve, S.; Kakodkar, S. Green synthesis of silver nanoparticles using cauliflower waste and their multifaceted applications in photocatalytic degradation of methylene blue dye and Hg2+ biosensing. SN Appl. Sci. 2020, 2, 738-789.
28. Ding, Y.; Wu, J.; Wang, J.; Wang, J.; Ye, J.; Liu, F. Superhydrophilic carbonaceous-silver nanofibrous membrane for complex oil/water separation and removal of heavy metal ions, organic dyes and bacteria. J. Membr. Sci. 2020, 614, 118-191.
29. El-Ghmari, B.; Farah, H.; Ech-Chahad, A. A New Approach for the Green Biosynthesis of Silver Oxide Nanoparticles Ag_2O, Characterization and Catalytic Application. Chem. React. Eng. Catal. 2021, 16, 456-478.
30. Bi, N.; Zheng, H.; Zhu, Y.; Jiang, W.; Liang, B. Visible-light-driven photocatalytic degradation of non-azo dyes over Ag_2O and its acceleration by the addition of an azo dye. J. Environ. Chem. Eng. 2018, 6, 3150-3160.
31. Xu, Z.; Zada, N.; Habib, F.; Ullah, H.; Hussain, K.; Ullah, N.; Bibi, M.; Bibi, M.; Ghani, H.; Khan, S.; et al. Enhanced Photocatalytic Degradation of Malachite Green Dye Using Silver–Manganese Oxide Nanoparticles. Molecules. 2023, 28, 245-287.
32. Aravind, M.; Amalanathan, M.; Mary, M.S.M. Synthesis of TiO_2 nanoparticles by chemical and green synthesis methods and their multifaceted properties. SN Appl. Sci. 2021, 3, 409-425.
33. Ngoepe, N.M.; Mathipa, M.M.; Hintsho-Mbita, N.C. Biosynthesis of titanium dioxide nanoparticles for the photodegradation of dyes and removal of bacteria. Optik. 2020, 224, 165-228.
34. Skiba, M.; Vorobyova, V.; Sorochkina, K. Bio-Green and Classical Stabilize Agents of Nanoparticles in Silver-Doped Titanium Dioxide: The Influence on Antioxidant, Photocatalytic, and Bactericidal Activities. Plasmonics. 2022, 17, 2221-2234.
35. Altaf, N.U.; Naz, M.Y.; Shukrullah, S.; Ghaffar, A.; Irfan, M.; Walczak, D.; Głowacz, A.; Mahnashi, M.H.; Rahman, S.; Królczyk, G.; Alqarni, A.O., Niazi, U.M. Concurrent Synthesis and Immobilization of Ag Nanoparticles over TiO2 via Plasma Reduction for Photocatalytic Treatment of Methyl Blue in Water. Materials. 2021, 14, 516-587.
36. Fouda, A.; Hassan, S.E.-D.; Abdel-Rahman, M.A.; Farag, M.M.S.; Shehal-deen, A.; Mohamed, A.A.; Alsharif, S.M.; Saied, E.; Moghanim, S.A.; Azab, M.S. Catalytic degradation of wastewater from the textile and tannery industries by green synthesized hematite (α-Fe_2O_3) and magnesium oxide (MgO) nanoparticles. Curr. Res. Biotechnol. 2021, 3, 29-41.
37. Bhuiyan, M.S.H.; Miah, M.Y.; Paul, S.C.; Aka, T.D.; Saha, O.; Rahaman, M.M.; Sharif, M.J.I.; Habiba, O.; Ashaduzzaman, M. Green synthesis of iron oxide nanoparticle using Carica papaya leaf extract: Application for photocatalytic degradation of remazol yellow RR dye and antibacterial activity. Heliyon. 2020, 6, 460-487.

38. Mohamed, H.S.; Soliman, N.K.; Moustafa, A.F.; Abdel-Gawad, O.F.; Taha, R.R.; Ahmed, S.A. Nano metal oxide impregnated Chitosan-4-nitroacetophenone for industrial dye removal. Int. J. Environ. Anal. Chem. 2021, 101, 1850-1877.
39. Fan, H.; Ma, X.; Zhou, S.; Huang, J.; Liu, Y.; Liu, Y. Highly efficient removal of heavy metal ions by carboxymethyl celluloseimmobilized Fe_3O_4 nanoparticles prepared via high-gravity technology. Carbohydr. Polym. 2019, 213, 39-49.
40. Ijimdiya, S.J.; Ijimdiya, R.U.; Ajala, A.; Abdullahi, N.I. A comprehensive review of the innovations, applications, and future prospects of nanomaterials in water treatment. Nano-Struct. Nano-Objects. 2025, 44, 101583.
41. Lagiewka, J.; Nowik-Zajac, A.; Pajdak, A.; Zawierucha, I. A novel multifunctional β-cyclodextrin polymer as a promising sorbent for rapid removal of methylene blue from aqueous solutions. Carbohydr. Polym. 2023, 307, 120615.
42. Yayayürük, A.E.; Çankaya, N.; Yayayürük, O. D-limonene-based polymeric adsorbent for effective Pb (II) ion removal: A comprehensive study on sorption behaviour and environmental applicability. Colloids Surf. A Physicochem. Eng. Asp. 2024, 694, 134104.
43. Wang, P.; An, G.; Jarvis, P.; Liu, W.; Ding, S.K.; Qu, R.X.; Li, Z.H.; Ye, C.; Chu, W.H. Simultaneous removal of organic micropollutants and metals from water by a multifunctional β-cyclodextrin polymer-supported-polyaniline composite. Chem. Eng. J. 2024, 482, 148826.
44. Raj, A.; Rego, R.M.; Ajeya, K.V.; Jung, H.Y.; Kurkuri, M.D. Basil seeds loaded with MOFs as an eco-friendly and sustainable adsorbent for efficient removal of hazardous organic pollutants from water. Sep. Purif. Technol. 2024, 330, 125370.
45. Zhang, Y.; Lei, Y.; Yan, T.; Liao, Y.; Han, G. Efficient adsorption removal of 2, 4-dichlorophenoxyacetic acid using amine-functionalized metal–organic frameworks (MOFs): performance and mechanisms. Sep. Purif. Technol. 2024, 334, 126120.
46. Akar, T.; Alim, S.; Meltem, G.; Sayin, F.; Akar, S.T. A novel sustainable and eco-friendly biosourced hybrid sorbent for toxic Pb^{2+} decontamination: Nano metal oxide functionalized salt-tolerant plant biomass. J. Clean. Prod. 439, 2024, 140838.
47. Ortiz-Bustos, J.; Soares, S.F.; del Pulgar, H.P.; Pérez, Y.; Gómez-Ruiz, S.; Daniel-da-Silva, A.L.; del Hierro, I. Tuning adsorption capacities of hybrid mesoporous silica nanospheres and adsorption mechanism study for sulfamethoxazole and diclofenac removal from water. J. Mol. Liq. 2024, 398, 124213.
48. Goncalves, G.; Domingues, E.M.; Ferreira, N.; Ranawadia, K.; Henriques, B.; Bessa, A.; Tavares, D.; Martins, N.; Pereira, E.; Marques, P.A.A.P. Enhanced Hg (II) removal using thiourea-functionalized graphene oxide: Lab to pilot scale evaluation. Sep. Purif. Technol. 2024, 351, 128053.
49. Geranmayeh, S.; Mastali, S.; Mohammadnejad, M. Functionalized iron Oxide/MOF nanocomposite exhibiting excellent performance for methylene blue adsorption. Inorg. Chem. Commun. 2024, 162, 112222.
50. Alghamdi, H.M.; Elgarahy, A.M.; Zoromba, M.S.; Elwakeel, K.Z. A microwave-regenerable multi-walled carbon nanotube/polyaniline/Fe_3O_4 ternary nanocomposite for quantifiable sorption of cationic and anionic dyes. Colloids Surf. A Physicochem. Eng. Asp. 2024, 698, 134438.
51. Meena, H.M.; Kukreti, S.; Jassal, P.S. Synthesis of a novel chitosan-TiO_2 nanocomposite as an efficient adsorbent for the removal of methylene blue cationic dye from wastewater. J. Mol. Struct. 2025, 1319, 139420.
